# CRISPR screens in iPSC-derived neurons reveal principles of tau proteostasis

**DOI:** 10.1101/2023.06.16.545386

**Published:** 2023-06-26

**Authors:** Avi J. Samelson, Nabeela Ariqat, Justin McKetney, Gita Rohanitazangi, Celeste Parra Bravo, Darrin Goodness, Ruilin Tian, Parker Grosjean, Romany Abskharon, David Eisenberg, Nicholas M. Kanaan, Li Gan, Carlo Condello, Danielle L. Swaney, Martin Kampmann

**Affiliations:** 1 Institute for Neurodegenerative Diseases, University of California, San Francisco, CA, USA; 2 University of California San Francisco, Quantitative Biosciences Institute (QBI), San Francisco, CA, USA; 3 University of California San Francisco, Department of Cellular and Molecular Pharmacology, San Francisco, CA, USA; 4 Gladstone Institute of Data Science and Biotechnology, Gladstone Institutes, San Francisco, CA, USA; 5 Helen and Robert Appel Alzheimer Disease Research Institute, Feil Family Brain and Mind Research Institute, Weill Cornell Medicine, New York, NY, USA; 6 Departments of Chemistry and Biochemistry and Biological Chemistry, UCLA-DOE Institute, UCLA, Los Angeles, CA USA; 7 Howard Hughes Medical Institute UCLA, Los Angeles, CA, USA; 8 Department of Translational Neuroscience, Michigan State University, East Lansing, MI, USA; 9 Department of Neurology, University of California San Francisco, San Francisco, ca; 10 Department of Biochemistry and Biophysics, University of California San Francisco, San Francisco, CA, USA

**Keywords:** neurodegeneration, proteostasis, protein aggregation, tau, CRISPR screen

## Abstract

A hallmark of age-associated neurodegenerative diseases is the aggregation of proteins. Aggregation of the protein tau defines tauopathies, which include Alzheimer’s disease and frontotemporal dementia. Specific neuronal subtypes are selectively vulnerable to the accumulation of tau aggregates, and subsequent dysfunction and death. The mechanisms underlying cell type-selective vulnerability are unknown. To systematically uncover the cellular factors controlling the accumulation of tau aggregates in human neurons, we conducted a genome-wide CRISPRi-based modifier screen in iPSC-derived neurons. The screen uncovered expected pathways, including autophagy, but also unexpected pathways including UFMylation and GPI anchor synthesis, that control tau oligomer levels. We identify the E3 ubiquitin ligase CUL5 as a tau interactor and potent modifier of tau levels. In addition, disruption of mitochondrial function increases tau oligomer levels and promotes proteasomal misprocessing of tau. These results reveal new principles of tau proteostasis in human neurons and pinpoint potential therapeutic targets for tauopathies.

## Introduction

Tauopathies, which include Alzheimer’s disease, are widespread neurodegenerative diseases defined by pathological aggregation of the protein tau and there are limited therapeutics for these diseases. Tau is an intrinsically disordered protein that has six splice isoforms and is known to interact with microtubules. More recent work has established the role of tau in diverse neuronal processes, including axonal transport and synaptic transmission^1–5^. Mutations in the gene that encodes tau, *MAPT*, cause familial forms of frontotemporal lobar degeneration (FTLD)^6–8^. Most tauopathies, however, are not familial^8,9^, suggesting that factors in the cellular environment contribute to tauopathy onset.

A key characteristic of tauopathies is selective vulnerability: specific regions of the brain and neuronal subtypes within them are vulnerable to specific tauopathies^10–13^, again pointing to the importance of the cellular environment for tau aggregation. Furthermore, recent structures of tau aggregates from patients have revealed disease-specific tau aggregate structures^14^, suggesting that determinants of tau conformation in different cellular environments may drive distinct pathological conformation and disease outcomes. Tau is also known to be highly post-translationally modified, and specific post-translational modifications are associated with disease^15,16^, providing a direct link between tau primary sequence and differential expression or activity of factors in the cellular environment.

A major challenge has been to identify the specific cellular factors contributing to early changes in tau conformation that can lead to tau misfolding and cellular toxicity and dysfunction. GWAS studies^17–20^ uncover modifiers of disease risk but do not provide molecular mechanisms. Similarly, single-cell transcriptomics^11,21,22^, can describe the factors differentially expressed in vulnerable versus resilient neuronal subtypes, but these studies lack direct experiments to pinpoint those factors that causally control pathology.

Experimental model systems enable the mechanistic dissection of factors controlling tau aggregation, but some previously described models have limitations in terms of physiological relevance (such as tau over-expression in non-neuronal cell types) or are not amenable to high-throughput functional experiments that are required to gain a comprehensive understanding of factors determining tau conformational control in human neurons.

Here, we use tau conformational-specific antibodies as probes for CRISPR-based genetic modifier screens in iPSC-derived human neurons^23^ harboring the mutation *MAPT* V337M, which is 100% penetrant for FTLD^24,25^. We utilize this new screening methodology to perform a genome-wide screen for tau oligomers. Tau oligomers are formed during the early stages of aggregation and there is some evidence that they are more toxic than larger protein aggregates^26–30^. We then perform several secondary screens to compare modifiers of tau levels and tau oligomers in isogenic pairs of iPSC-derived neurons harboring the *MAPT* V337M and WT genotypes.

We find that the strongest single gene shared among these screens, *CUL5*, is a new tau interactor that controls tau levels. We follow up on the single strongest class of genes–those genes involved in mitochondrial function– and find that acute oxidative stress induces a proteasome-derived tau fragment. This fragment is very similar to many tauopathy cerebrospinal fluid (CSF) biomarkers, suggesting that these biomarkers could be a signature of oxidative stress induced proteasome dysfunction. Together, these data provide a comprehensive and unbiased view of cellular factors that control tau levels and oligomerization in human neurons.

## Results

### MAPT V337M neurons accumulate tau oligomers

**Figure 1:**
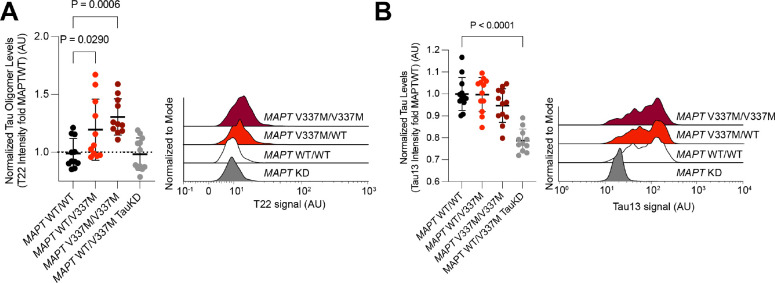
*MAPT V337M* neurons have higher levels of tau oligomers. **(A)** *Left,* Tau oligomer levels measured using the antibody T22 by flow cytometry in isogenic iPSC-derived neurons with one or two copies of the dementia-causing *MAPT* V337M mutation. *MAPT* knockdown neurons (grey) are included to establish background fluorescence signal. Intensities were normalized to the average of WT tau neurons (black). *Right,* Representative histograms are shown. **(B)** *Left,* Tau levels measured using the total tau antibody tau13 by flow cytometry in the same panel of neurons. Intensities were normalized to the average of WT tau neurons (black). *Right,* representative histograms are shown. Two-way ANOVA was used for all statistical analysis. All samples are the average of twelve biological replicates, error bars are ±standard deviation.

Since iPSC-derived models of tauopathy do not spontaneously accumulate mature pathological forms of tau, such as neurofibrillary tangles, we decided to characterize early stages of aggregation. Specifically, tau oligomers that can be detected by conformation-specific antibodies. We compared the binding of conformation-specific antibodies between isogenic *MAPT* WT (hereafter referred to as WT) and *MAPT* V337M (hereafter referred to as V337M) iPSC-derived neurons at 14 days post-differentiation. Tau oligomer levels were higher in V337M neurons than WT neurons, as measured by the oligomer-specific antibody T22^29,30^ using flow cytometry (Figure 1A–B). Importantly, knockdown of *MAPT* using CRISPRi reduced levels of T22 staining, confirming the dependence of T22 levels on tau expression. Furthermore, we observed a dose-dependent effect of T22 staining dependent on the expression of one (*MAPT* WT/V337M) or two (*MAPT* V337M/V337M) copies of the V337M tau variant.

### Genome-wide screen for modifiers of tau oligomer levels

**Figure 2:**
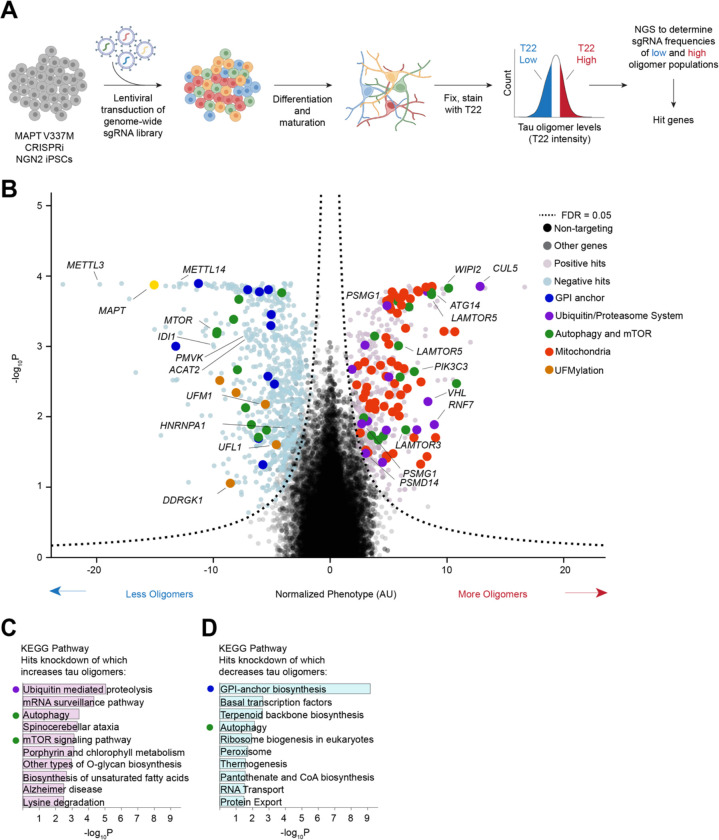
Genome-wide screen for tau oligomers levels in iPSC-derived neurons. **(A)** Schematic of screen. Tau V337M heterozygous iPSCs were transduced with a pooled library of sgRNAs targeting every protein-coding gene. iPSCs were differentiated into excitatory neurons for two-weeks, fixed, and stained with T22 and NeuN. The thirty percent of NeuN-positive cells with the lowest (blue) and highest (red) tau oligomer signal were separated by FACS sorting. Genomic DNA was isolated from each population and the sgRNA cassette was sequenced using next-generation sequencing. Comparison of sgRNA frequencies was used to call hit genes. **(B)** Volcano plot of hit genes from genome-wide screen. Phenotype (normalized log2 of T22 High versus T22 low counts), is plotted versus the negative log of the P-value, calculated with a Mann-Whitney U-test. Positive hits are in pink, and negative in light blue. Non-targeting controls are in black and non-hit genes are in grey. *MAPT* is a top hit (yellow). Groups of genes as identified by KEGG Pathway analysis **(C)** are highlighted: GPI-anchor (blue), Ubiquitin Proteasome System (Purple), Autophagy and mTOR (green), Mitochondrial genes (red), and UFMylation genes (orange). **(C)** KEGG Pathway analysis was performed after removal of mitochondrial genes. Top ten pathways by adjusted p-value are listed for hits knockdown of which increase tau oligomer levels (light pink) and for hits knockdown of which decrease tau oligomer levels (light blue).

To enable a genome-wide modifier for screen for tau oligomer levels in V337M neurons, we engineered the V337M iPSC line to express CRISPRi machinery and optimized a protocol (see methods) for the FACS sorting of fixed iPSC-derived neurons using the anti-tau oligomer antibody T22 (Figure 2A). Briefly, we infected iPSCs with a lentiviral CRISPRi sgRNA library targeting all protein coding genes (five sgRNAs per gene, 104,535 sgRNAs) and 1,895 non-targeting controls^31^, differentiated them into neurons, and fixed them at Day 14 post-differentiation. Neurons were then stained with T22 antibody and an antibody against the neuronal marker NeuN. NeuN-positive cells were FACS sorted into two bins: those neurons that had the highest and the lowest thirty percent of T22 signal. Frequencies of sgRNAs in each bin were determined by next-generation sequencing and compared to NTCs using a Mann-Whitney U test to assign p-values for each gene (Figure 2B). 1,143 genes were called hits based on a false discovery rate (FDR) of 5% (see Methods).

Knockdown of *MAPT* was a top hit that decreased tau oligomer levels, as expected. Pathway analysis of hit genes revealed several pathways expected based on the previous literature. Known regulators of tau and tau oligomer levels, such as genes that control autophagy, were significantly enriched (Figure 2B–D), with knockdown of the autophagic machinery and positive regulators of autophagy, including *WIPI2, ATG14, PIK3C3, ATG101,* and *LAMTOR1,3* and *5* increasing tau oligomer levels, while knockdown of negative regulators of autophagy, such as *MTOR*, decreased tau oligomer levels^32–35^. Recent studies have implicated methyl-6-Adenosine (m^6^A) as a modulator of tau oligomerization^36^ and knockdown of genes involved in the regulation and deposition of m^6^A, including *METTL14, METTL3,* and *HNRNPA1*, were among the tops hits that decrease tau levels upon knockdown. These results confirm that the methodology used here recapitulates shared characteristics of tau biology in cell culture, mouse models, and human samples.

The strongest gene signature overall in this screen are genes involved in mitochondrial function, especially the electron transport chain (ETC). Knockdown of these genes strongly increased tau oligomer levels (Fig. 2B). KEGG pathway analysis without mitochondrial hits (Figure 2C) revealed other pathways controlling tau oligomer levels. As mentioned above, knockdown of positive regulators of autophagy and lysosomal degradation were significantly enriched as top hits that increase tau oligomer levels. Factors from the other branch of protein degradation, the Ubiquitin/Proteasome System (UPS) were also among the top hits in this screen, including *VHL*, the proteasome chaperones *PSMG1* and *PSMG3*, the proteasomal deubiquitinase *PSMD14* (also known as rpn11), and several E3, E2, and E1 ubiquitin ligases. The Cullin-RING ligase (CRL) *CUL5*, and its obligate E2 ligase adaptor, *RNF7*, were also among top hits knockdown of which increases tau oligomer levels.

KEGG pathway analysis of genes knockdown of which decreased tau oligomer levels included negative regulators of autophagy, including the genes that encode the mTORC1 subunits mTOR, RAPTOR, and MLST8, which inhibit autophagy through ULK1. Surprisingly, GPI-anchor biosynthesis was the most significantly enriched set of genes whose knockdown decreases tau oligomer levels. Knockdown of genes including *ACAT2*, *PMVK*, *IDI1*, and *FDPS*, which are involved in synthesis of mevalonate, a precursor to molecules such as cholesterol, also decreased tau oligomer levels. Finally, knockdown of genes essential for UFMylation, including *UFM1*, *UFL1*, and *DDRGK1*, decreased tau oligomer levels. In a related manuscript, we validate UFMylation as a pathway controlling levels of tau aggregates^37^.

### Secondary screens pinpoint cellular factors controlling tau levels and genotype-specific factors

**Figure 3:**
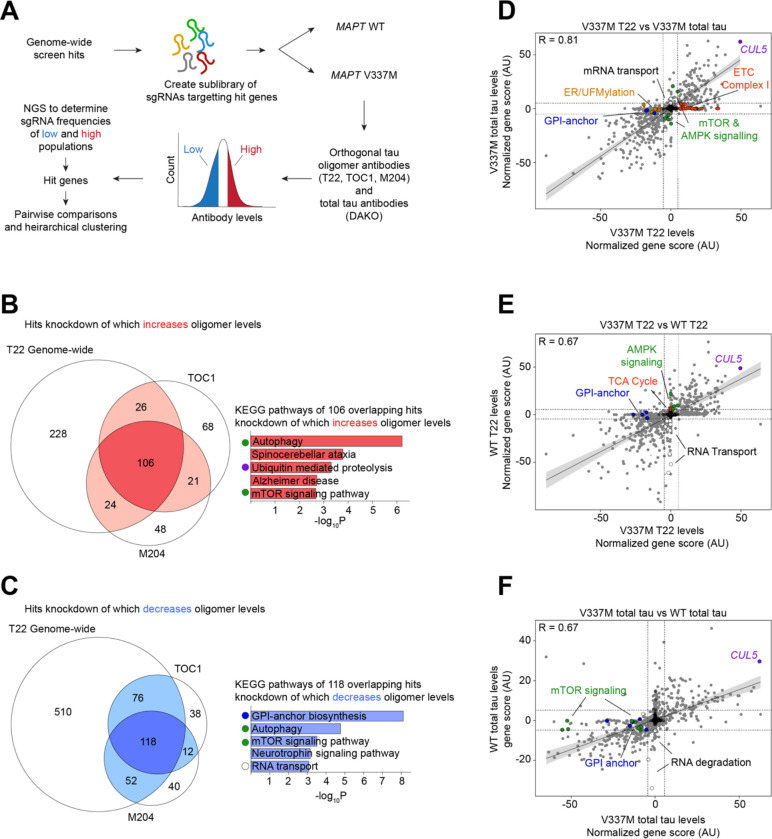
Secondary screens reveal modifiers specific to tau oligomer levels and MAPT genotype **(A)** Schematic of retest strategy. A focused sgRNA library targeting all the hits from the genome-wide screen was screened in *MAPT* WT and V337M neurons using a panel of different antibodies. **(B)** Overlapping hit genes knockdown of which increases tau oligomer levels between three tau oligomer antibodies, T22, TOC1, and M204. Top KEGG Pathways of overlapping genes are listed after removal of mitochondrial genes. **(C)** Overlapping hit genes knockdown of which decreases tau oligomer levels between three tau oligomer antibodies, T22, TOC1, and M204. Top KEGG Pathways of the overlapping genes are listed. **(D)** Comparison of total tau and tau oligomer screens in V337M neurons by gene score. ETC Complex I genes (red), ER/UFMylation genes (orange), and GPI-anchor genes (blue) were oligomer specific hits. *CUL5* is in purple. mTOR and AMPK signaling genes are in green. **(E)** Comparison of WT and V337M tau oligomer screens by gene score. GPI-anchor genes (blue) were V337M specific, while knockdown of genes involved in mRNA transport (white), especially nuclear pore subunits, strongly decreased tau oligomer levels in WT but not V337M neurons. Knockdown TCA cycle genes (red) and genes involved in AMPK signaling (green) weakly increased tau oligomers in WT, but not V337M neurons. *CUL5* is in purple. **(F)** Comparison of screens for total tau levels in MAPT V337M versus WT neurons. V337M total tau levels were more sensitive to knockdown of mTOR signaling (green) and GPI anchor genes (blue). WT total tau levels were more sensitive to knockdown of genes involved in RNA degradation (white). *CUL5* is in purple.

To characterize the top hits more closely, we cloned a small, pooled library targeting 1,037 hit genes (5 sgRNAs per gene) and 250 non-targeting controls and performed seven CRISPRi secondary screens using this library (Figure 3A).

The first three screens used two alternative tau oligomer antibodies, TOC1^38^ and M204^39^, as well as retesting T22. There was a substantial overlap in modifiers of tau oligomers detected by all three antibodies. KEGG Pathway analysis of the 106 shared hits knockdown of which increased oligomer levels detected by all three antibodies confirmed that autophagy, mTOR signalling, and the UPS system are high-confidence gene sets that control tau oligomer levels (Figure 3B–C). Conversely, GPI-anchor biosynthesis and RNA transport were pathways enriched among genes knockdown of which decreases tau oligomer levels detected by all three antibodies (Figure 3C).

**Figure 4:**
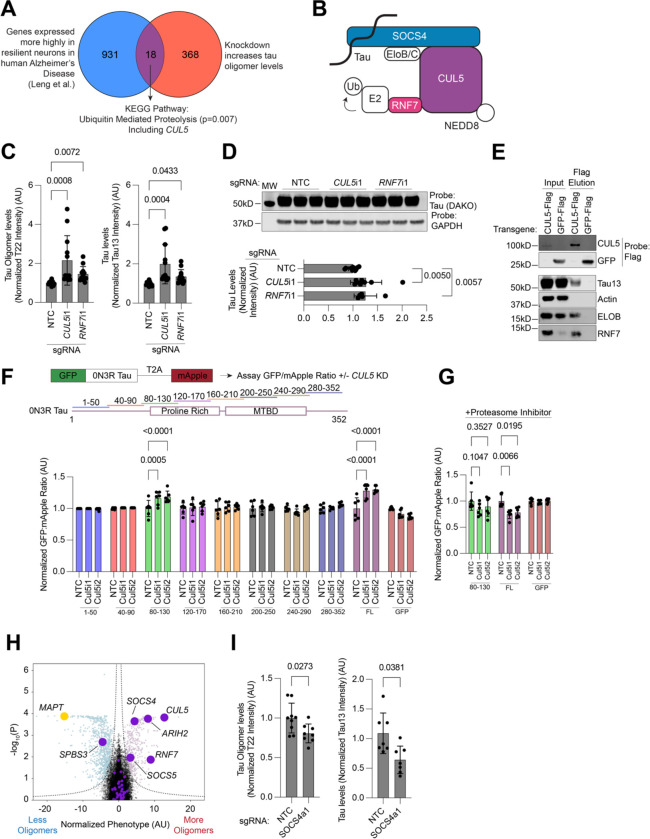
CUL5 regulates tau levels. **(A) C**omparison between genes expressed more highly in excitatory neurons in the human entorhinal cortex that are resilient versus vulnerable to Alzheimer’s diseases ^11^ and genes that increase tau oligomer levels in this study. **(B)** Schematic of CUL5 E3 Ligase complexes. **(C,D)** Individual knockdown of CUL5 and RNF7 reveals increases in Tau oligomer and total tau levels by flow cytometry (B) and western blot (C). Average of twelve biological replicates **(E)** CUL5-flag co-elutes with tau, as well as other members of CUL5 complexes, elongin B (ELOB) and RNF7. **(F)** Ratiometric reporter assay for determining CUL5-dependent degrons in tau (top). Constructs tiling the tau sequence are shown in different colors, with amino acid numbers referring to the 0N3R sequence (middle). Results from the ratiometric reporter in cells expressing non-targeting (NTC) sgRNAs or sgRNAs targeting Cul5. Only tau itself and tile 3 (residues 80–130) increase in levels upon *CUL5* knockdown and this effect is abrogated upon treatment with the proteasome inhibitor carfilzomib at 10nM **(G)**. **(H)** Volcano plot as in Figure 2, but with all known *CUL5* adaptors colored in purple, as well as the *CUL5*, *RNF7*, and the *CUL5* interactor *ARIH2*. **(I)** Tau oligomer (left) and total tau levels (right) measured during overexpression of SOCS4 using CRISPRa. Average of seven biological replicates. Two-way ANOVA was used for all statistical analysis. All data points are the average of six biological replicates, unless otherwise stated. Error bars are ±standard deviation.

We next screened both the WT and V337M lines using a total tau antibody, and the WT line using T22, to find hits that uniquely control tau overall or tau oligomer levels, as well as hits unique to V337M, respectively (Figure D-F). ETC complex I hits where unique to the tau oligomer screens, as were GPI-anchor biosynthesis hits and ER/UFMylation genes. The WT tau line seemed to be more sensitive to knockdown of genes involved in mRNA transport, while the V337M line was enriched for hits that regulate mTOR. Finally, the E3 ubiquitin ligase CUL5 was a top hit in all screens we performed regardless of tau mutation or antibody. Intriguingly, CUL5 is also a gene expressed more highly in excitatory neurons in the human entorhinal cortex that were resilient to Alzheimer’s disease, compared to vulnerable neurons in a scRNA-seq study performed by our lab^11^ (Fig. 4A), suggesting that it may causally contribute to selective vulnerability in the context of Alzheimer’s disease.

### CUL5 controls tau levels via tau ubiquitination

CUL5 is a Cullin-RING (CRL) E3 ubiquitin ligase that is best known for its role in regulating aspects of viral infection, especially for HIV^40–42^. CUL5 serves as a scaffold for facilitating ubiquitin transfer from an E2 ligase to CUL5 substrates. RNF7, also a strong hit in our screen (see Figure 2B), is essential for binding of E2s to CUL5. The N-terminal region of CUL5 binds to substrates via modular substrate adaptors that are bound to CUL5 with the proteins Elongin B and Elongin C (ELOB and ELOC, also known as TCEB2 and TCEB3) (Figure 4A). Activity of CUL5 is dependent on neddylation, a ubiquitin-like protein that is conjugated by the NEDD8-specific E2 UBE2F.

Knockdown of both *CUL5* and *RNF7* with independently cloned sgRNAs recapitulated screen phenotypes by flow cytometry and western blot (Figure 4C–D). Western blot showed smaller increases in tau levels, possibly because flow cytometry only measures tau levels in the neuron soma and not in axons. We then over-expressed CUL5–3xFLAG to ask if CUL5 and tau physically interact. Immunoprecipitation of CUL5–3xFLAG, but not GFP-3xFLAG, and subsequent FLAG elution revealed tau in the eluate (Figure 4E). Importantly, ELOB and RNF7 were both present in the eluate, confirming that functional CUL5 complexes were immunoprecipitated.

Cullin-ring ligases (CRLs) are known to recognize substrates via degrons, short amino acid sequences that bind to CRL substrate adaptors. To find the region of tau that acts as a CUL5 degron, we created N-terminal GFP fusions of eight tau “tiles” using a sliding window of fifty amino acids with a ten amino acid overlap and fused them to a T2A-mApple sequence to create a ratiometric reporter of tau tile levels (Figure 4F). We made stable lines expressing these tiles in iPSCs using lentivirus, transduced them with sgRNAs against *CUL5* or non-targeting controls, differentiated them into neurons and, 14 days post-differentiation, assayed the GFP/mApple ratio. Along with full length tau, only Tile 3, 0N3R tau residues 80–130 (2N4R tau residues 138–188) is sensitive to *CUL5* knockdown. This increase in Tile 3 levels upon *CUL5* knockdown was dependent on proteasome activity, as treatment with the proteasome inhibitor Carfilzomib abrogated this effect (Figure 4G).

CRLs bind substrates via adaptor proteins that bind to the N-terminal region of the cullin. In our screen, two CUL5 adaptors were positive hits- SOCS4 and SOCS5 (Figure 4H). Overexpression of SOCS4 using CRISPRa decreased tau levels, suggesting that CUL5 binds tau via SOCS4 (Figure 4I).

### Oxidative stress promotes the formation of a proteasome-derived N-terminal tau fragment

Genes essential for the function of mitochondria, as well as other genes such as *FECH, PSAP* and *FH,* knockdown of which we previously found to increase levels of reactive oxygen species (ROS) in neurons^43^ (Figure S1) were all hits knockdown of which increased tau and tau oligomer levels in our primary and secondary screens. We validated these hits using a pharmacological approach to investigate the role mitochondrial function, especially the ETC, on tau oligomer levels.

**Figure 5:**
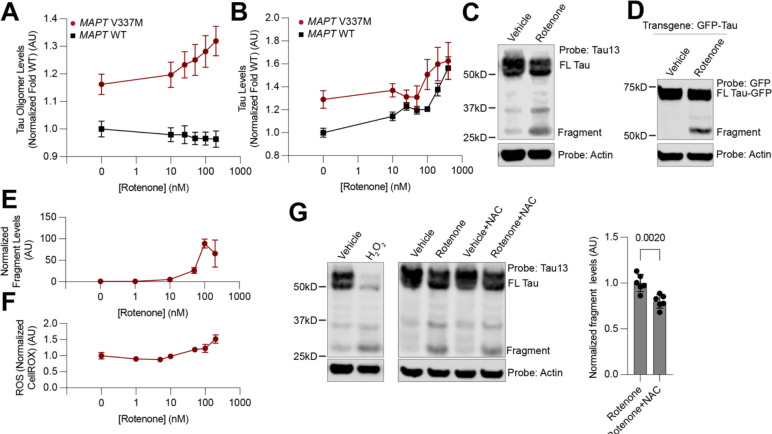
Oxidative stress increases tau oligomer and total tau levels and induces a tau cleavage fragment. **(A,B)** Levels of tau oligomers (A) and total tau (B) increase in a rotenone concentration-dependent manner in V337M but only tau levels, and not tau oligomer levels increase in WT tau neurons. **(C)** Western blot of rotenone-treated neurons reveals a tau cleavage fragment. **(D)** Rotenone treatment of neurons results in an equivalent fragment from a GFP-Tau transgene. Fragment levels as measured by western blot **(E)** and ROS levels as measured by flow cytometry using CellROX **(F)** show similar rotenone concentration-dependent increases. **(G)** Treatment with 100μM hydrogen peroxide (right) drastically increases fragment formation, while treatment with the antioxidant N-Acetyl-Cysteine at 1μM (right) decreases fragment formation (quantitation far right). If not otherwise shown, rotenone concentration is at 200nM. Two-way ANOVA was used for all statistical analysis. All samples are the average of six biological replicates, error bars are ±standard deviation.

We treated neurons with increasing concentrations of rotenone, an ETC complex I inhibitor, for 24 hours and measured tau and T22 levels by flow cytometry (Figure 5A–B). This revealed a rotenone concentration-dependent increase in both tau and T22 levels in the V337M line. Therefore, all further experiments were done in the V337M line. Validation of these phenotypes by Western blot revealed that rotenone promoted the formation of a ~25kD fragment of tau detected by the tau13 antibody, which recognizes tau residues 2–18 (Figure 5C). Expression of a GFP-0N3R tau transgene and treatment with 200nM rotenone revealed the same molecular weight shift (Figure 5D), confirming that this fragment is due to post-transcriptional processing of tau.

Measurement of ROS levels using CellRox, and 25 kD-fragment levels, by western blot, as a function of rotenone concentration revealed a concentration-dependent increase similar to that of T22 signal and tau levels (Figure 5E–F). Treatment with antimycin A, an ETC complex III inhibitor, but not CCCP, a proton-gradient uncoupler, or Oligomycin, an ATP Synthase inhibitor, also led to 25 kD-fragment formation (Figure S2), suggesting that ROS production as a side effect of mitochondrial dysfunction induces 25 kD-fragment formation. Neurons treated with hydrogen peroxide revealed close to complete conversion of full-length tau into the 25 kD-fragment (Figure 5G). Concomitant treatment of neurons with rotenone and N-acetyl-cysteine, an antioxidant, decreased 25 kD-fragment levels as compared to rotenone alone (Figure 5G). Thus, acute increases in ROS levels generate this tau proteolytic fragment. 25 kD-fragment formation was not due to cell death via apoptosis or ferroptosis, as treatment with the pan-caspase inhibitor ZVAD-MVK, the ferroptosis inhibitor ferrostatin, or the ferroptosis inducer RSL3 did not change fragment levels (Figure S3). This experiment also rules out caspase cleavage of tau to generate the 25kD-fragment).

**Figure 6:**
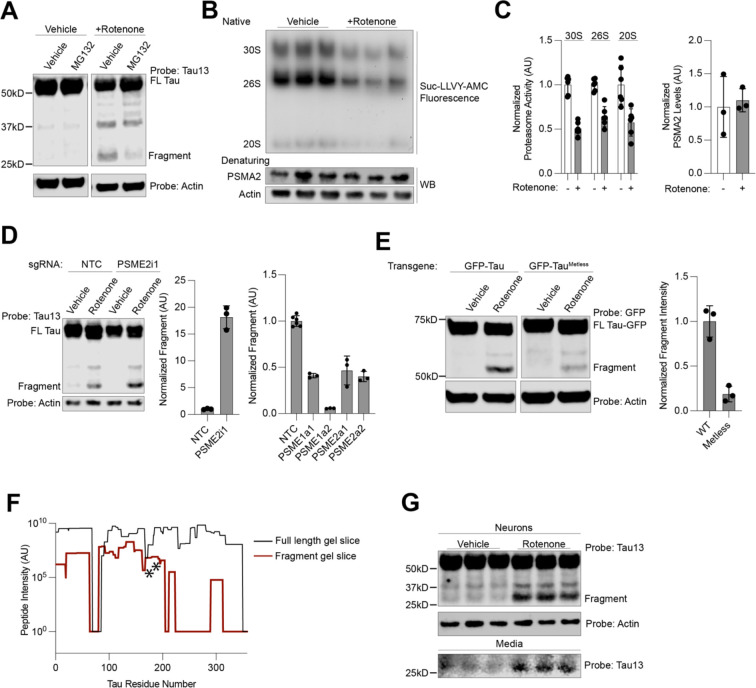
Proteasome dysfunction causes accumulation of the tau cleavage fragment. **(A)** Treatment with proteasome inhibitor MG132 at 1μM decreases levels of the 25kD rotenone-induced tau fragment. **(B)** Treatment with rotenone decreases proteasome activity (top) but not levels (bottom). **(C)** Quantitation of **(B)**, average of six biological replicates. **(D)** Knockdown of PSME2 increases fragment levels (left) Western blot with quantitation. Over-expression with CRISPRa of *PSME1* and *PSME2* decrease fragment levels (right). Full gels in Figure S6. **(E)** Expression of GFP-tau^Metless^ decreases fragment formation as compared to GFP-tau in neurons. Western blot (left), quantitation of western blot (right). **(F)** Traces of averaged intensity from peptides per amino acid derived from mass spectrometry data for full-length tau fragment (black) and rotenone induced fragment (dark red). A sharp decrease in intensity is seen in the rotenone induced fragment gel slice after residue 200, except for one peptide (see Figure S7). Stars represent neo-tryptic termini identified upon digestion with GluC, pinpointing two possible C-termini for the fragment, narrowing the fragment identity to residues 172–200 (2N4R numbering: 230–258). **(G)** Western blot of cell lysate (top) and undiluted neuron-conditioned media (bottom) shows fragment in the media. Rotenone concentration in all figures is 200nM. All data contain the average of three biological replicates, unless otherwise stated. Error bars are ±standard deviation.

We next treated neurons with 200nM rotenone and different protease inhibitors to identify if any cellular proteases induced this cleavage fragment. We chose inhibitors targeting common tau proteases: cathepsins, calpain, and the proteasome^44–47^ (Figure S4). Only inhibition of the proteasome decreased 25 kD-fragment formation (Figure 6A), suggesting that proteasomal processing or misprocessing creates this N-terminal tau fragment.

Proteasome activity, measured by native gel, of neurons treated with rotenone revealed a fifty percent decrease in activity of 30S, 26S and 20S proteasomes (Figure 6B–C), although no changes in levels of proteasomal proteins were observed. Thus, we hypothesize that changes in proteasome processivity, rather than levels, may promote formation of the proteolytic tau fragment.

Upon induction of oxidative stress, the proteasome activator PA.28 is often up-regulated or increases its association with the 20S proteasome in order to deal with increased proteostatic load due to the oxidation of proteins^48,49^. Overexpression by CRISPRa of the two PA.28 subunits, PA.28α and PA.28β, encoded by the genes *PSME1* and *PSME2* respectively, decreased 25-kD fragment formation (Figure 6D, left). Knockdown of the PA.28 subunit PA.β increased fragment levels dramatically (Figure 6D, right), further suggesting that it is the inability of the proteasome to processively degrade oxidized proteins that leads to fragment accumulation.

We next hypothesized that direct tau oxidation of tau leads to fragment formation. 0N3R tau only has one cysteine, C291, which is located close to the microtubule-binding domain. Expression of a GFP-0N3R tau transgene with the cysteine mutated to alanine (C291A) did not change fragment levels compared to a transgene with the cysteine (Figure S4). Therefore, we hypothesized that oxidation of tau’s six methionines, 5 of which are in the first 75 residues of tau, could be directly oxidized upon induction of oxidative stress and thus lead to proteasome misprocessing. We cloned a methionine-free version of tau, tau^Metless^, where every methionine is mutated to leucine. This construct, although expressed at the same levels as WT tau, showed a large decrease in fragment formation (Figure 6E). Taken together, we hypothesize that direct oxidation of tau by ROS allows the proteasome to aberrantly engage with and proteolyze tau, leaving the observed 25kD N-terminal fragment.

We then over-expressed GFP-Tau and purified the fragment for identification by mass spectrometry (Figure 6F). We observed a substantial decrease in the observance and intensity of peptides at approximately the 200th residue of the 0N3R tau sequence. However, considering that we did not identify any non-tryptic peptides in that region that would serve as the C-terminus for a fragment digested by an endogenous protease, we hypothesized that the digestion may be performed by a trypsin-like protease, consistent with trypsin-like activity of the proteasome. This led us to pursue our second strategy, an in-solution digest of GFP-tau purified from neurons that had been treated with rotenone using the protease GluC. After GluC digestion and LC-MS/MS, we identified three peptides that were semi-specific for GluC with tryptic N-termini, ending at 172 and 176 in the 0N3R MAPT sequence. Thus, the fragment sequence is narrowed to a small region spanning residues 172–200 in this region (2N4R tau residues 230–258). This is remarkably similar to the tau biomarker peptides present in tauopathy patient CSF, which end near residue 230, and tau fragments previously identified in iPSC-neuron conditioned media^50–52^. ELISA-based CSF tauopathy biomarker assays that use an N-terminal antibody and phospho-tau epitopes 181 or 231 are the most accurate for disease prediction^51^. We then asked whether the 25kD-fragment could be secreted from neurons. Indeed, we were able to detect its presence in the conditioned media of neurons, which increased upon rotenone treatment (Figure 6G). Thus, we hypothesize that N-terminal tau biomarkers could be markers of neuronal oxidative stress and resulting changes in proteasome activity.

## Discussion

Here we established that *MAPT* V337M iPSC-derived neurons have higher levels of tau oligomers than *MAPT* WT iPSC-derived neurons. We performed a genome-wide CRISPRi-screen to uncover cellular factors controlling tau oligomer accumulation. To complement this primary we screen, we performed several small-scale CRISPRi screens using orthogonal tau oligomer specific antibodies and total tau antibodies in both the *MAPT* V337M and *MAPT* WT backgrounds. This strategy uncovered known modulators of tau and tau oligomer levels, validating this approach, and revealed novel regulators of tau and tau oligomer levels, such as *CUL5*.

In comparison to other tau screens in the literature^53–56^, our screen adds a disease-relevant context for tau genetic modifier screens: human iPSC-derived neurons. In combination with our use of conformational-specific antibodies, we are able to discriminate between those genes affecting only tau levels or only tau misfolding. We performed screens in both mutant (V337M) and wildtype tau genotypes and cross-compared these screens for factors specific to genotype and antibody used.

Generally, pathways identified in our screens agree with those previously identified in the literature. Novel pathways elucidated in our screens, such as GPI-anchor proteins or UFMylation, may be neuron-specific, as the currently published large-scale screens were performed in cancer cell lines. A previous screen performed in SHY5Y cells^55^, identified *CUL5* knockdown as a negative modifier of tau levels, contrary to our work, which identifies *CUL5* knockdown as a positive modifier of tau levels. Our earlier finding that CUL5 is expressed more highly in human entorhinal cortex neurons resilient to Alzheimer’s disease than in those that are selectively vulnerable^11^ highlights CUL5 as a potential causal determinant of selective vulnerability in human brains. Further work will need to be done to identify the mechanisms by which CUL5 regulates tau levels.

Knockdown of nuclear-encoded mitochondrial genes is the primary signature of the genome-wide screen presented here, and knockdown of those genes increases tau oligomer levels. This signature adds to a growing body of literature that connects tau function, mitochondrial function, oxidative stress, and neurodegenerative disease^57–60^. Mechanistic studies here reveal one possible mechanism by which CSF-based biomarkers for tauopathies may be generated. We found that ETC inhibition generates acute oxidative stress, which in turn leads to proteasome misprocessing of oxidized tau. The resultant protein fragments are very similar in sequence to known CSF biomarkers^50–52^. There are other possible mechanisms by which mitochondrial dysfunction may also relate to tau misfolding or dysfunction. For instance, knockdown of ETC complex I genes has recently been recognized to increase tolerance for cellular ROS. More mechanistic work must be done to fully elucidate the relationship between tau and mitochondrial biology.

## STAR★Methods

### RESOURCE AVAILABLILITY

#### LEAD CONTACT AND MATERIALS AVAILABILITY

The cell lines generated in this study are available on request upon the completion of a Material Transfer Agreement (MTA). All plasmids generated in this study will be deposited on AddGene. Further information and requests for resources and reagents should be directed to and will be fulfilled by the Lead Contact, Martin Kampmann (martin.kampmann@ucsf.edu).

#### DATA AND CODE AVAILABILITY

- CRISPR screening data have been deposited at CRISPR-brain (https://www.crisprbrain.org/) and will be publicly available as of the date of publication.
- CRISPR screening raw data will be made available upon request
- All original code is available at https://kampmannlab.ucsf.edu/scripts-tau-oligomer-screen-manuscript.

### EXPERIMENTAL MODEL AND SUBJECT DETAILS

#### Human iPSCs

Human iPSCs (in the male WTC11 background (Miyaoka et al., 2014) were cultured in StemFlex Medium on BioLite Cell Culture Treated Dishes (Thermo Fisher Scientific; assorted Cat. No.) coated with Growth Factor Reduced, Phenol Red-Free, LDEV-Free Matrigel Basement Membrane Matrix (Corning; Cat. No. 356231) diluted 1:100 in Knockout DMEM (GIBCO/Thermo Fisher Scientific; Cat. No. 10829–018). Routine passaging was performed as described ^23^. Studies with human iPSCs at UCSF were approved by the The Human Gamete, Embryo and Stem Cell Research (GESCR) Committee. Informed consent was obtained from the human subjects when the WTC11 (Miyaokaet al., 2014) lines were originally derived.

#### Human iPSC-derived neurons

Human iPSC-derived neurons were pre-differentiated and differentiated as described ^23^. Briefly, iPSCs were pre-differentiated in Matrigel-coated plates or dishes in N2 Pre-Differentiation Medium containing the following: KnockOut DMEM/F12 as the base, 1× MEM non-essential amino acids, 1× N2 Supplement (Gibco/Thermo Fisher Scientific, cat. no. 17502–048), 10 ng ml^−1^ of NT-3 (PeproTech, cat. no. 450–03), 10 ng ml^−1^ of BDNF (PeproTech, cat. no. 450–02), 1 μg ml^−1^ of mouse laminin (Thermo Fisher Scientific, cat. no. 23017–015), 10 nM ROCK inhibitor and 2 μg ml^−1^ of doxycycline to induce expression of mNGN2. After 3 d, on the day referred to hereafter as Day 0, pre-differentiated cells were re-plated into BioCoat poly-D-lysine-coated plates or dishes (Corning, assorted cat. no.) in regular neuronal medium, which we refer to as +AO neuronal medium, containing the following: half DMEM/F12 (Gibco/Thermo Fisher Scientific, cat. no. 11320–033) and half neurobasal-A (Gibco/Thermo Fisher Scientific, cat. no. 10888–022) as the base, 1× MEM non-essential amino acids, 0.5× GlutaMAX Supplement (Gibco/Thermo Fisher Scientific, cat. no. 35050–061), 0.5× N2 Supplement, 0.5× B27 Supplement (Gibco/Thermo Fisher Scientific, cat. no. 17504–044), 10 ng ml^−1^ of NT-3, 10 ng ml^−1^ of BDNF and 1 μg ml^−1^ of mouse laminin. Neuronal medium was half-replaced every week. Full protocols are available on protocols.io (dx.doi.org/10.17504/protocols.io.bcrjiv4n).

#### HEK293T

HEK293Ts were cultured in DMEM supplemented with 10%FBS, 1% Pen/Strep, 1% Glutamine (DMEM complete). Cells were passaged by washing with DPBS, adding trypsin for 2 minutes at 37C, and quenching and resuspended in a 5-fold trypsin volume of DMEM complete. Cells were spun at 200×g for 5 minutes, counted, and plated at the desired density.

## METHOD DETAILS

### Generation of V337M CRISPRi line

WTC11 WT/V337M iPSCs harboring a single-copy of doxycycline-inducible mouse NGN2 at the AAVS1 locus^62^ were used as the parental iPSC line for further genetic engineering. iPSCs were transfected with pC13N-dCas9-BFP-KRAB and TALENS targeting the human CLYBL intragenic safe harbor locus (between exons 2 and 3) (pZT-C13-R1 and pZT-C13-L1, Addgene #62196, #62197) using DNA In-Stem (VitaScientific). After 14 days, BFP-positive iPSCs were isolated via FACS sorting, and individualized cells were plated in a serial dilution series to enable isolation of individual clones under direct visualization with an inverted microscope in a tissue culture hood via manual scraping. Clones with heterozygous integration of dCas9-BFP-KRAB (determined using PCR genotyping) were used for further testing. Full protocols are available on protocols.io: https://dx.doi.org/10.17504/protocols.io.8dahs2e.

### Flow cytometry

#### Fixed neurons

For CRISPR-screening, iPSC-differentiated neurons were washed with HBSS and then dissociated from the plate using Papain solution (20 U/mL papain in HBSS) at 37C for 10 minutes. Papain was quenched with 3x volume DMEM with 10% FBS and spun down 200×g for 10 minutes. Cells were then fixed with zinc fixation buffer (0.1M Tris-HCl, pH 6.5 @ 4C, 0.5% ZnCl2, 0.5% Zn Acetate, 0.05% CaCl2) overnight at 4C. When ready for staining, samples were washed three times in TBS by centrifugation at 200×g for 10 minutes and resuspended in permeabilization buffer (10% Normal Goat Serum, 10% 10x TBS, 3% BSA, 1% Glycine, 0.5% Tween-20) and blocked for 30 minutes. During blocking, cells were triturated into a single-cell suspension with progressively smaller pipette tips, from P1000 pipette tips to P20. After addition of primary antibodies, the antibody/cell slurry was incubated for two hours. Samples were then spun down at 200×g for 10 minutes and washed 3x with TBS by centrifugation. The cell pellet was then resuspended in permeabilization buffer with secondary antibodies and incubated at room temperature for one hour. Samples were then spun down at 200×g for 10 minutes and washed 3x with TBS by centrifugation. In the last wash, Hoechst at a concentration of 2 μM was added to the wash buffer for 10 minutes at room temperature. After the last wash, cells were resuspended in 1 mL of TBS and FACS sorted on a BD ARIA Fusion.

All other iPSC-differentiated neurons were prepared for flow cytometry in 96-well plate format as follows. iPSC-differentiated neurons were washed with TBS and then fixed with zinc fixation buffer at 4 °C overnight. When ready for staining, samples were washed three times in TBS and then 50uL of permeabilization buffer was added and incubated for 30 minutes to block. Primary antibody in permeabilization buffer was then added and samples incubated at 4 °C overnight. Primary was removed and cells were washed 4x in TBS. Secondary antibodies were added in permeabilization buffer and incubated for one hour at room temperature. Cells were washed 4x in TBS with Hoechst in the second to last wash. After the last wash, cells were triturated by pipetting up and down with a P200 tip 10 times and then a P20 tip 10 times and analyzed with a BD Celesta cell analyzer and the data were processed using FlowJo v10 software.

#### Live neurons

iPSC-differentiated neurons were washed with HBSS and dissociated from the plate using Papain solution (20 U/mL papain in HBSS) at 37 °C for 10 minutes. Papain was quenched with 3x volume DMEM with 10% FBS and spun down 200×g for 10 minutes. Cells were then resuspended in HBSS and analyzed with a BD Celesta analyzer the data were processed using FlowJo v10 software. For cells analyzed with CellRox (thermo), CellRox diluted to 5 μM in differentiation media was added 1:1 with the extant well media volume and incubated for 30 minutes at 37 °C. Cells were washed with HBSS three times and dissociated and flowed as described above.

### Lentivirus generation

#### sgRNAs

Lentivirus was generated as described ^23^. Briefly, HEK293T were plated to achieve 80–95% confluence 24 hours after plating. For a 6-well plate, 1ug of transfer plasmid, and 1ug of third generation packaging mix, were diluted into 200 μL OPTIMEM and 12 μL of TRANSIT-LENTI was added. Transfection mix was incubated at room temperature for 10 minutes and then added to HEKs. After 48 hours, supernatant was transferred to a syringe and filtered through a 0.45 μm PVDF filter into a conical tube. ¼ volume of Lentivirus Precipitation Solution was added to the filtrate and stored at 4 °C for 24 hours. Lentivirus-containing supernatant was then centrifuged for 30 min at 1500×g at 4 °C, supernatant aspirated, and then spun again at 4 °C for 5 mins at 1500×g. Supernatant was aspirated and the virus-containing pellet was resuspended in 200 μL DPBS. For screening, each library was prepared from a 15cm plate of HEK293Ts, scaled appropriately. For infection, virus was added at the same time as iPSC passaging. After 48 hours, cells were passaged, analyzed for marker positivity by flow cytometry and selected with 1 μg/mL puromycin until >95% marker positive, for two passages. Cells were allowed to recover for one passage before pre-differentiation. The full protocol is available on protocols.io (https://dx.doi.org/10.17504/protocols.io.8dfhs3n)

#### Over-expression constructs

Lentivirus was generated as described ^23^. Briefly, HEK293T were plated to achieve 80–95% confluence 24 hours after plating. For a 6-well plate, 1 μg of transfer plasmid, and 1 μg of third-generation packaging mix, were diluted into 200uL OPTIMEM and 12 μL of TRANSIT-LENTI was added. Transfection mix was incubated at room temperature for 10 minutes and then added to HEK293T cells. After 48 hours, supernatant was transferred to a syringe and filtered through a 0.45 μm PVDF filter into a conical tube. ¼ volume of Lentivirus Precipitation Solution was added to the filtrate and stored at 4 °C for 24 hours. Lentivirus-containing supernatant was then centrifuged for 30 min at 1500×g at 4 °C, supernatant aspirated, and then spun again at 4 °C for 5 mins at 1500×g. Supernatant was aspirated and the virus-containing pellet was resuspended in 200n μL DPBS. Cells were expanded to T75 flasks and when 80% confluent sorted for the appropriate marker (mApple or GFP).

### CRISPRi screening

For each genome-wide sub-library and each secondary screen, 45 million iPSCs in 3x T175s were infected with lentivirus as above at an MOI of ~0.3 and selected. Cells were then differentiated as above and plated on 3× 15-cm PDL-coated dishes at a density of 15 million cells per plate. Cells were then matured for two weeks and prepared for FACS sorting as above, staining for NeuN and the tau-specific antibodies as indicated. For the tau-specific antibodies with mouse host, NeuN staining was not performed. Cells were collected into 1mL of 30% BSA in a FACS tube. After sorting, cells were pelleted at 200×g for 20 minutes, the supernatant was removed and the pellet was frozen at −20. Genomic DNA was extracted with the NucleoSpin Blood L kit. sgRNA cassettes were amplified pooled and sequenced as described ^23^. Sequencing was analyzed as described for each sub-library ^23^. Screening data is available in Supplemental Tables 2 and 3.

### Cloning of secondary screen library

A pool of sgRNA-containing oligonucleotides were synthesized by Agilent Technologies and cloned into our optimized sgRNA expression vector as previously described^63^.

### Western Blotting

iPSC-derived neurons were cultured as described above. Neurons were washed 3x with ice-cold DPBS and then ice-cold RIPA with protease and phosphatase inhibitors was added to cells (50 μL for a 24-well plate). Lysates were incubated on ice for 2 minutes and then scraped down. Lysates were either flash frozen on liquid nitrogen or directly centrifuged at 21000×g for 10 minutes at 4 °C. The supernatants were then collected, and concentrations assessed using a BCA assay (Thermo). 10 μg protein were loaded onto a 4–12% Bis-Tris polyacrylamide gel (Thermo). Nitrocellulose membranes were used to transfer the protein in a BioRad Transblot for 11 minutes at 25 V, 25 A. Membranes were then blocked for 1 hour with Licor Odyssey block and primary was added in Licor Odyssey block overnight at 4 °C. Blots were then washed 4× 5 minutes with TBST and secondary antibodies were added in Licor Odyssey block for 1 hour at room temperature. Blots were washed 4× 5 minutes with TBST and imaged on a Licor. Immunoblots were quantified by intensity using ImageStudio (Licor).

### Immunoprecipitations

#### Flag IPs

iPSC-derived neurons were cultured as described above. Neurons were washed 3x with ice-cold DPBS and then lysed in FLAG-lysis buffer (20 mM HEPES NaOH pH 7.4, 150 mM NaCl, 0.2% NP40 with protease and phosphatase inhibitors and 2 μM 1,10-phenathroline). Lysates were freeze/thawed on liquid nitrogen 7 times and then centrifuged at 21000×g for 30 minutes at 4C. The supernatants were then collected and concentrations assessed using a BCA assay. 5 mg of lysate was loaded onto 25 μL FLAG dynabeads (thermo) that had been washed 3x with FLAG-lysis buffer. IP was performed with rotation at 4 °C for 2 hours. Beads were then washed 3x with FLAG-lysis buffer and then 2x with TBS. FLAG-peptide elution was performed with 1 mg/mL FLAG-peptide in TBS overnight at 4C. Acid elution with 100 mM glycine pH 2.0 was performed for 10 min and the supernatant was quenched in 1/10^th^ volume of 1M Tris pH 9.0. High-temperature elutions were performed using 2x LDS with 20 mM DTT for 70 °C for 5 minutes.

#### GFP IPs

iPSC-derived neurons were cultured as described above. Neurons were washed 3x with ice-cold DPBS and then lysed in GFP-lysis buffer supplemented with protease and phosphatase inhibitors (ProteinTech). Lysates were freeze/thawed on liquid nitrogen 7 times and then centrifuged at 21000×g for 30 minutes at 4 °C. The supernatants were then collected and diluted using GFP dilution buffer supplemented with protease and phosphatase inhibitors (ProteinTech). Concentrations assessed using a BCA assay. 100 mg of lysate was loaded onto 100 μL GFP-trap beads (ProteinTech) that had been washed 3x with Dilution buffer supplemented with protease and phosphatase inhibitors. IP was performed with rotation at 4 °C for 1 hours. Beads were then washed 3x with GFP-wash buffer and then 2x with TBS. Acid elution with 100 mM glycine pH 2.0 was performed for 10 min and the supernatant was quenched in 1/10^th^ volume of 1M Tris pH 9.0. Eluates were then loaded on a gel and excised for mass spectrometry (below).

### In-gel proteasome assay

Proteasome activity from neurons was measured as described (10.1016/j.xpro.2021.100526). Briefly, iPSC-derived neurons were washed 3x with ice cold DPBS, and then scraped into TSDG lysis buffer (10 mM Tris, 1.1 mM MgCl2, 10 mM NaCl, 1 mM NaN3, 1 mM DTT, 2 mM ATP, 10% glycerol) and lysed by freeze/thaw on liquid nitrogen 7 times. Samples were then spun down 10 minutes at 21000×g, assayed by BCA, and 50 μg supernatant was mixed with 5x native gel loading buffer (0.05% bromophenol blue, 43.5% glycerol, 250 mM Tris pH 7.5) to a concentration of 1x loading buffer. Samples were loaded on a 3–8% Tris Acetate gel (Thermo) was run in native gel running buffer (1x TBE, 413 μM ATP, 2 mM MgCl2, 0.5 mM DTT) for 3 hours at 170 V. Gels were then incubator for 30 min at 37 °C in reaction buffer (50 mM Tris pH 7.5, 10 mM MgCl2, 1 mM ATP, 1 mM DTT, 48 μM Suc-LLVY-AMC) and imaged on a Gel Doc EZ (Bio-rad).

### Mass spectrometry

Gel bands were then excised and the proteins making up each band were reduced, alkylated, and digested with trypsin all within the gel. The resulting peptides were then extracted from the gel slice into solution and analyzed using liquid chromatography tandem mass spectrometry (LC-MS/MS) with data-dependent acquisition. Preparation in solution was used because alternate proteases, such as GluC, cannot penetrate the gel matrix making gel separation and preparation infeasible. After acquiring and analyzing our LC-MS/MS data, we identified three peptides that were semi-specific for the GluC with tryptic N termini, ending at 172 and 176 in the fetal MAPT sequence.

### qPCR

RNA was extracted using the Zymo Quick-RNA miniprep kit and cDNA was synthesized with the SensiFAST cDNA synthesis kit. Samples were prepared for qpCR in technical triplicates using SensiFAST SYBR Lo-ROX 2x Mastermix. qPCR was performed on an Applied Biosystems Quantstudio 6 Pro Real-Time PCR System using Quantstudio Real Time PCR Software following Fast 2-Step protocol: (1) 95 °C for 20 s; (2) 95 °C for 5 s (denaturation); (3) 60 °C for 20 s (annealing/extension); (4) repeat steps 2 and 3 for a total of 40 cycles; (5) 95 °C for 1 s; (6) ramp 1.92 °C s^−1^ from 60 °C to 95 °C to establish melting curve. Expression fold changes were calculated using the ΔΔCt method, normalizing to housekeeping gene *GAPDH*. Primer sequences are provided in Table S1. Quantification of qPCR samples are shown in Figure S7.

### Drug treatments

At the two-week feeding, 50% of the media volume was removed and drugs diluted in media were added in order to obtain the correct drug concentration when adding media to reach the previous media volume.

## QUANTIFICATION AND STATISTICAL ANALYSIS

For statistical analysis, we used GraphPad Prism 9.5.1. Data are shown as mean±SD, except for flow cytometry data, which is shown as the median±SD. For two sample comparions, un unpaired two-tailed Student’s t-test was used. For three sample comparison, a two-way ANOVA was used. P-values are shown above compared samples, n.s. denotes not significant.

### CRISPR-screen analysis

#### Primary screen analysis

CRISPR screens were analyzed using MAGeCK-iNC as previously described ^23^. Briefly, raw sequencing reads were cropped and aligned using custom scripts that are already publicly available (https://kampmannlab.ucsf.edu/resources). Raw phenotype scores and p-values were calculated for target genes and negative control genes using a Mann-Whitney U-test. Hit genes were identified using a FDR of 0.05. Gene scores were normalized by the standard deviation of negative controls genes for each genome-wide sublibrary. Hits were then combined and gene set enrichment analysis (GSEA) was performed for T22 positive and negative bins after filtering for mitochondrial genes using ENRICHR^64–66^.

#### Pairwise analysis secondary screens

After data processing as described above, normalized hits files for re-test screens were compared using custom python scripts to calculate Pearson’s correlation coefficient and generate lists of genes that were unique to each screen in the comparison using a gene score of ±5. We then performed GSEA using ENRICHR on these unique gene sets in order to label categories of genes. Venn diagrams of overlapping hits were generated using a custom python script and the list of overlapping genes processed for GSEA using ENRICHR, after filtering for mitochondrial genes.

## Supplementary Material

Supplement 1

Supplement 2

Supplement 3

Supplement 4

Supplement 5

## Figures and Tables

**KEY RESOURCES TABLE:** 

REAGENT or RESOURCE	SOURCE	IDENTIFIER
**Antibodies**		
Rabbit anti-Actin	Cell Signaling	Cat#4970
Mouse anti-GFP	Santa Cruz Biotech	Cat#9996
Rabbit anti-tau oligomer (T22)	Rakez Kayed, The University of Texas Medical Branch at Galveston	Gift
Rabbit anti-tau oligomer (M204)	David Eisenberg, University of California, Los Angeles	Gift
Mouse anti-tau oligomer (TOC1)	Nicholas Kanaan, Michigan State University	Gift
Mouse anti-tau (Tau13)	Santa Cruz Biotech	Cat#21796
Mouse anti-NeuN	Biolegend	Cat#SIG-39860
Mouse anti-Rbx2 (RNF7)	Santa Cruz Biotech	Cat#166554
Rabbit anti-ELOB	ProteinTech	Cat#A0024
Rabbit anti-tau	DAKO	Cat#05-803
Mouse anti-PSMA2	Thermo Fisher Scientific	Cat#MA5-26149
**Biological samples**		
**Chemicals, peptides, and recombinant proteins**		
StemFlex Media	Thermo Fisher Scientific	Cat# A3349401
Y-27632	Stem Cell Technologies	Cat#72308
Matrigel hESC-Qualified Matrix	Corning	Cat#354277
Doxycycline hyclate (Dox)	Sigma-Aldrich	Cat#D9891
Brain-derived neurotrophic factor (BDNF)	PeproTech	Cat#450-02
Neurotrophin-3 (NT-3)	PeproTech	Cat#450-03
KnockOut DMEM/F-12	Thermo Fisher Scientific	Cat#12660012
Neurobasal A	Thermo Fisher Scientific	Cat#A3582901
DMEM/F-12	Thermo Fisher Scientific	Cat#11320033
GlutaMAX Supplement	Thermo Fisher Scientific	Cat#35050061
MEM Non-Essential Amino Acids Solution (100X)	Thermo Fisher Scientific	Cat#11140050
StemPro Accutase	Thermo Fisher Scientific	Cat#A1110501
N-2 Supplement	Thermo Fisher Scientific	Cat#17502048
B-27 Plus Supplement	Thermo Fisher Scientific	Cat#A35828-01
DPBS, no calcium, no magnesium	Thermo Fisher Scientific	Cat#14190144
Protease Inhibitor Cocktail	Millipore Sigma	Cat#11836170001
Nitrocellulose membrane	Biorad	Cat#1620115
Hanks’ Balanced Salt Solution (HBSS)	Sigma-Aldrich	Cat#H9394
Trypsin-EDTA (0.05%), phenol red	Thermo Fisher Scientific	Cat#25300054
Opti-MEM I Reduced Serum Medium	Thermo Fisher Scientific	Cat#31985070
Papain, Lyophilized	Worthington Biochemical	Cat#LS003118
TransIT-Lenti Transfection Reagent	Mirus Bio	Cat#MIR6600
Lentivirus Precipitation Solution	Alstem	Cat#VC100
Puromycin	Sigma	Cat#P9620
Bovine Serum Albumin	Sigma Aldrich	Cat#A7906-100G
Pierce Anti-DYKDDDK Magnetic Agarose	Thermo Fisher Scientific	Cat#A36797
Pierce Anti-DYKDDDK Magnetic Agarose	Thermo Fisher Scientific	Cat#A36805
SensiFAST cDNA Synthesis Kit	Meridian Bioscience	Cat#BIO65054
SensiFAST SYBR Lo-Rox	Meridian Biosceince	Cat#BIO94050
CellROX Green Reagent	Thermo Fisher Scientific	Cat#C10444
Succ-LLVY-AMC	Cayman Chemical	Cat#10008119
Licor Intercept Blocking Buffer	LI-COR	Cat#927-60001
Lipofectamine Stem	Thermo Fisher Scientific	Cat#STEM00003
**Critical commercial assays**		
P3 Primary Cell 4D-Nucleofector^™^ X Kit S	Lonza	Cat#V4XP-303
Pierce BCA Protein Assay Kit	Thermo Fisher Scientific	Cat#23225
QuickRNA MicroPrep Kit	Zymo	Cat#R1051
Zymo Gigaprep kit	Zymo	Cat#D4204
Poly-D-Lysine Culture Dishes	Corning	Cat#354550
Universal Mycoplasma Detection Kit	ATCC	Cat#30-1012K
**Deposited data**		
Raw and processed data (bulk CRISPR-screen data)	This paper	
**Experimental Models: Cell Lines**		
HEK293T	ATCC	Cat#CRL-11268
Human WTC11 iPSC with Ngn2 transgene and CRISPRi integration	Tian et al., 2019	N/A
Human WTC11 iPSC with Ngn2 transgene and CRISPRa integration	Tian et al., 2022	N/A
Human WTC11 iPSC with Ngn2 transgene and CRISPRi integration WT/V337M	This paper	N/A
Human WTC11 iPSC with Ngn2 transgene integration V337M/V337M	Sohn et al. 2019^61^	N/A
**Oligonucleotides**		
Primers for sequencing; see Table S1	This paper	N/A
Primers for qPCR; see Table S2	This paper	N/A
**Recombinant DNA**		
pMD2.G	Didier Trono	Addgene #12259
pMDLg/pRRE	Didier Trono	Addgene #12251
pRSV-REV	Didier Trono	Addgene #12253
pC13N-CLYBL-CAG-dCas9-BFP-KRAB	Martin Kampmann	Addgene #127968
pMK1334	Martin Kampmann	Addgene #127965
pAJS1197	This paper	N/A
pAJS1203	This paper	N/A
pAJS1205	This paper	N/A
pAJS1206	This paper	N/A
pAJS1207	This paper	N/A
pAJS1208	This paper	N/A
pAJS1209	This paper	N/A
pAJS1210	This paper	N/A
pAJS1211	This paper	N/A
pAJS1212	This paper	N/A
pAJS1213	This paper	N/A
pAJS1184	This paper	N/A
pAJS1154	This paper	N/A
pAJS1149	This paper	N/A
pZTC-13-R1	Jizhong Zou	Addgene #62196
pZT-C13-L1	Jizhong Zou	Addgene #62197
**Software and Algorithms**		
Adobe Illustrator	Illustrator v26.5.2	https://www.adobe.com/products/illustrator.html
GraphPad Prism 9	Prism v9.2	https://www.graphpad.com
FlowJo	FlowJo v10	https://www.flowjo.com/
MAGeCK-iNC	Tian et al., 2019	https://kampmannlab.ucsf.edu/mageck-inc
Imagestudio	LICOR	https://www.licor.com/bio/image-studio/
