# Supplementary material for: CRISPR screens in iPSC-derived neurons reveal principles of tau proteostasis
Source: bioRxiv. 2024 Oct 26:2023.06.16.545386. Preprint. [Version 3] doi: 10.1101/2023.06.16.545386 (PMC10312804; doi:10.1101/2023.06.16.545386)
Supplement: Supplementary file 1 [file NIHPP2023.06.16.545386v3-supplement-7.pdf]

bioRxiv preprint doi: https://doi.org/10.1101/2023.06.16.545386; this version posted October 26, 2024. The copyright holder for this preprint (which was not certified by peer review) is the author/funder, who has granted bioRxiv a license to display the preprint in perpetuity. It is made available under aCC-BY 4.0 International license.

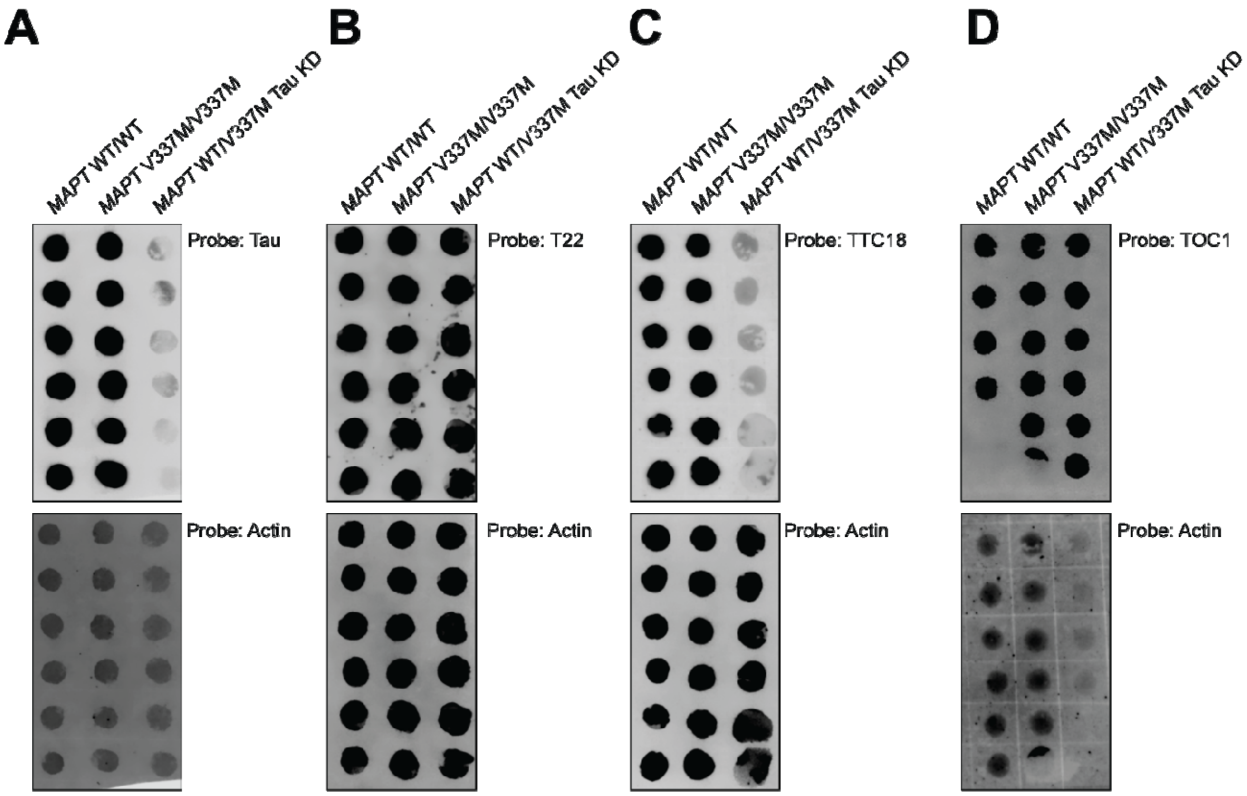

Figure S1: Dot blots used for quantitation in Figure 1C. Top: Blots probed with either (A) Tau13 (B) T22 (C) TTC18 or (D) TOC1. Below: Blots probed with Actin as a loading control.

bioRxiv preprint doi: https://doi.org/10.1101/2023.06.16.545386; this version posted October 26, 2024. The copyright holder for this preprint (which was not certified by peer review) is the author/funder, who has granted bioRxiv a license to display the preprint in perpetuity. It is made available under aCC-BY 4.0 International license.

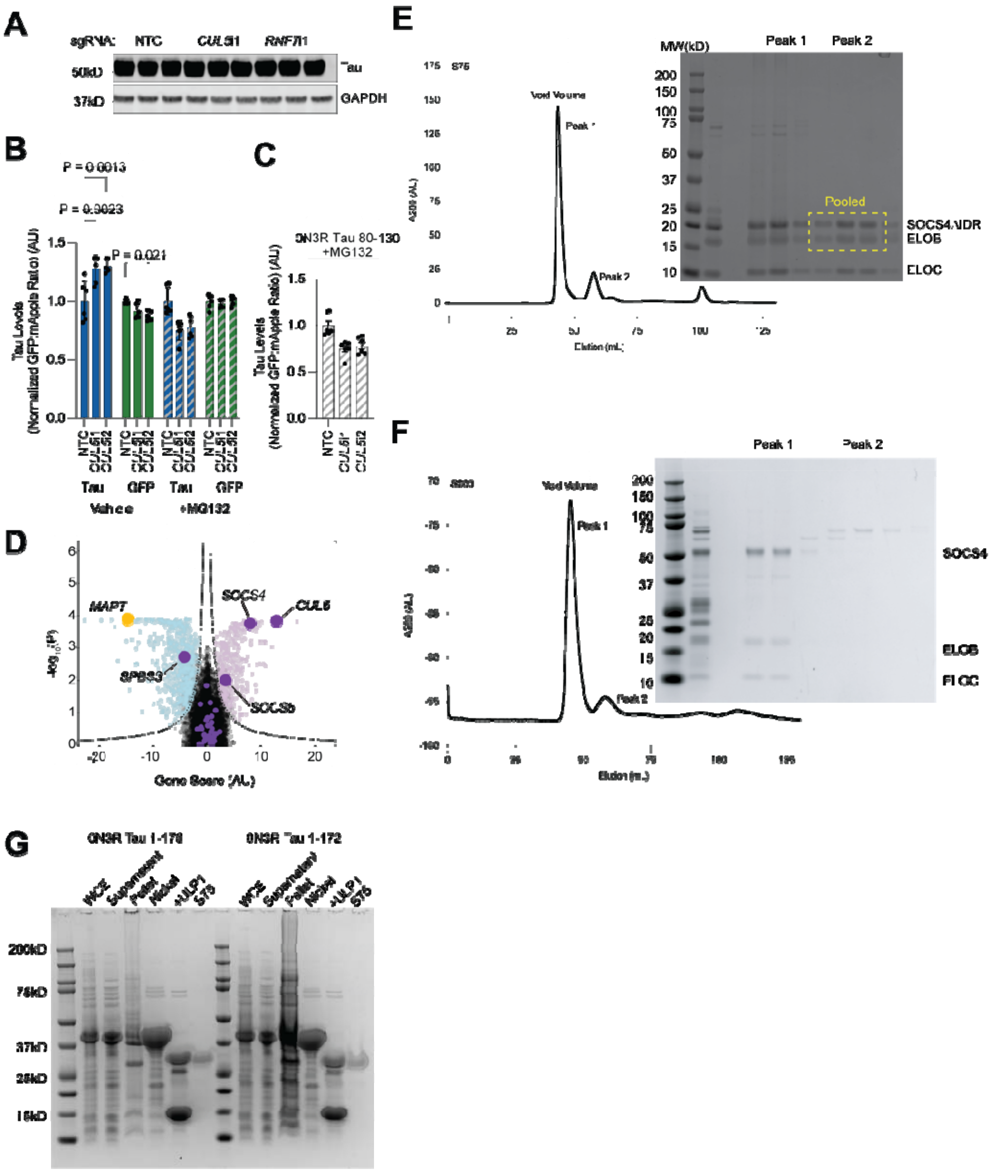

Figure S2: Related to Figure 4 and 7. (A) Western blots used in Figure 4B. **(B)** *CUL5* knockdown increases tau levels (blue), but does not increase GFP levels (b*ottom*). Six biological replicates were used per sample. This effect is rescued by treatment with the proteasome inhibitor MG132 (grey patterned bars). **(C)** Treatment of cells expression tau 80-130 with or without *CUL5* knockdown in the presence of the proteasome inhibitor MG132 shows a rescue of *CUL5* KD as compared to vehicle. Six biological replicates were used per sample. For all applicable subpanels, one-way ANOVA was used for statistical analysis. P-values of >0.05 are not shown. Error bars are ±standard deviation. **(D)** Volcano plot of hits from the genome-wide CRISPRi screen for modifiers of tau oligomer levels as in Figure 2, but with all known CRL5 adaptors colored in purple. Only *SOCS4* and, to a lesser degree, *SOCS5* knockdown increase tau oligomer levels. *CUL5* is labeled for reference. **(E)** Chromatogram trace of SOCS4ΔIDR-ELOBC (*left*) and SDS-PAGE of selected peaks (*right*). **(F)** Chromatogram trace of SOCS4 full length (*left*) and SDS-PAGE of selected peaks (*right*). **(G)** Purification of tau 25kD fragments.

bioRxiv preprint doi: https://doi.org/10.1101/2023.06.16.545386; this version posted October 26, 2024. The copyright holder for this preprint (which was not certified by peer review) is the author/funder, who has granted bioRxiv a license to display the preprint in perpetuity. It is made available under aCC-BY 4.0 International license.

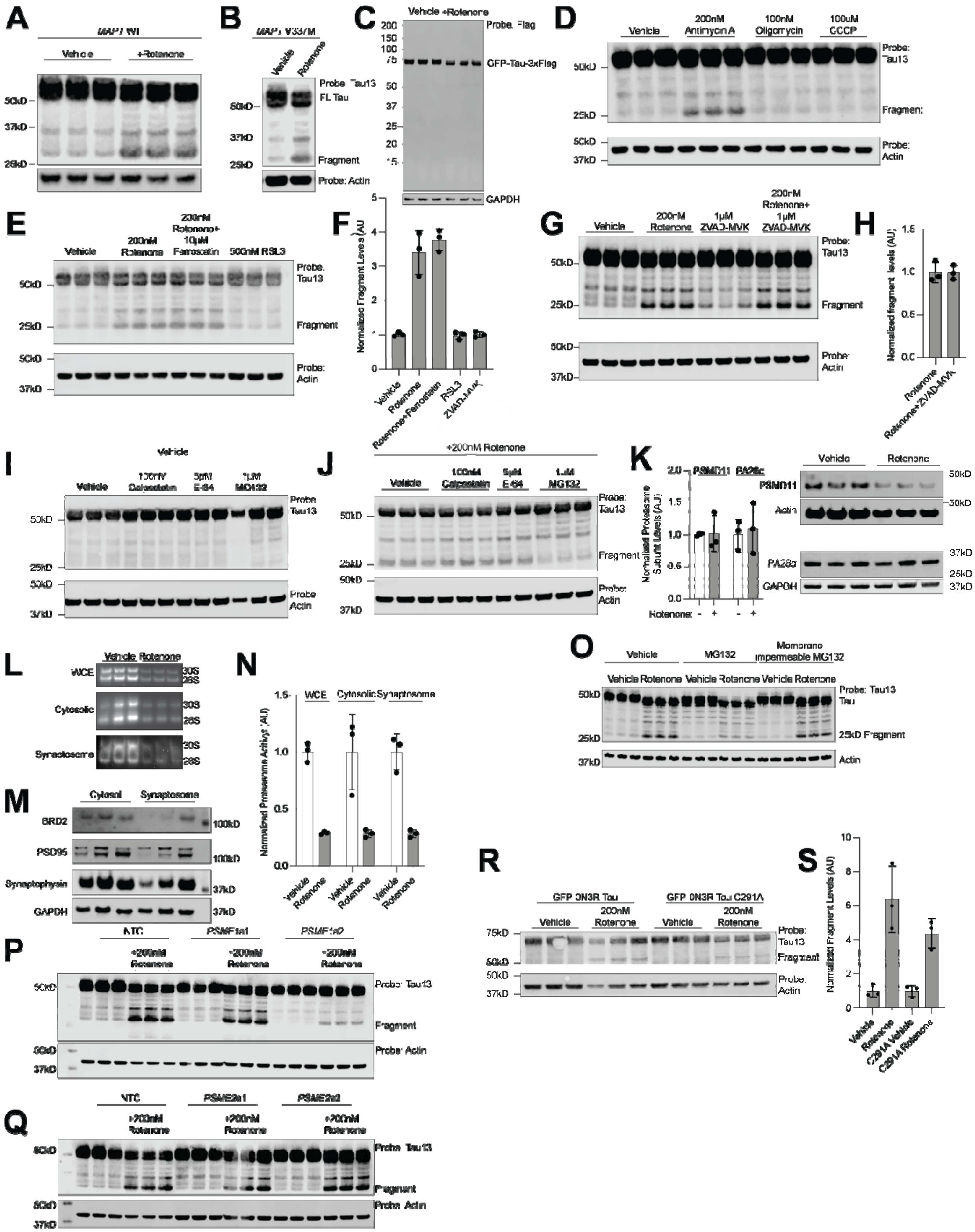

Figure S3: Rotenone treatment leads to 25kD fragment formation. (A) Biological triplicate

bioRxiv preprint doi: https://doi.org/10.1101/2023.06.16.545386; this version posted October 26, 2024. The copyright holder for this preprint (which was not certified by peer review) is the author/funder, who has granted bioRxiv a license to display the preprint in perpetuity. It is made available under aCC-BY 4.0 International license.

wells of *MAPT* WT neurons treated with vehicle (DMSO) or 200nM rotenone for 24 hours. **(B)** Figure 6C from the main text for comparison with the MAPT V337M tau line. **(C)** Neurons expressing a GFP-0N3R Tau-3xFlag construct treated with vehicle or 200nM rotenone for 24 hours. Probe with an anti-Flag antibody shows no low molecular weight tau fragments. **(D)** Inhibition of ETC complex III, but not complex V or uncoupling of the mitochondrial protein gradient promotes fragment formation**.** Western blot of vehicle treated, antimycin A treated (complex III inhibitor), oligomycin (complex V inhibitor) or CCCP (protein gradient uncoupler). **(E-H)** Apoptosis and ferroptosis do not control 25kD fragment formation. **(E)** Western blot of rotenone-treated neurons with the ferroptosis inhibitor ferrostatin, or ferroptosis promoting molecule RSL3 reveals no changes to tau 25kD fragment. **(F)** Quantitation of data in (E). **(G)**Western blot of rotenone-treated neurons with the pan-caspase inhibitor ZVAD-MVAK reveals no changes to tau 25kD fragment. **(H)** Quantitation of data in (C). **(I)-(J)** Proteasome inhibition decreases 25kD fragment formation. **(I)** Western blot of neurons with the cathepsin inhibitor E64, calpain inhibitor calpastatin, and proteasome inhibitor MG132. **(J)** Western blot of rotenone treated neurons with the cathepsin inhibitor E64, calpain inhibitor calpastatin, and proteasome inhibitor MG132. **(K)** Levels of PSMD11 and PA28[illegible] do not change upon rotenone treatment. Quantitation (*left*) of western blots (*right*). **(L-M)** Synaptic proteasome activity is decreased to the same extent as the entire proteasome pool. **(L)** Proteasome activity assay of proteasomes derived from whole cell lysate or the cytosolic and synaptosome fraction respectively. **(N)** Quantitation of activity from gels in (L)**. (N)** Synaptosome prep reveals depletion of nuclear proteins (BRD2) and enrichment of synaptic proteins. **(O)** Treatment with membrane-impermeable proteasome inhibitor does not affect 25kD fragment formation. **(P-Q)** Gels quantified in Figure 6 **(P)** CRISPRa of PSME1. **(Q)** CRISPRa of PSME2. **(R-S)** Mutation of Tau C291 does not affect fragment formation. **(R)** Western blot of neurons transduced with GFP-tau or GFP-tau C291A treated with rotenone or vehicle. **(S).** Quantitation of data in (R). All samples are the average of three biological replicates, error bars are ±standard deviation.

bioRxiv preprint doi: https://doi.org/10.1101/2023.06.16.545386; this version posted October 26, 2024. The copyright holder for this preprint (which was not certified by peer review) is the author/funder, who has granted bioRxiv a license to display the preprint in perpetuity. It is made available under aCC-BY 4.0 International license.

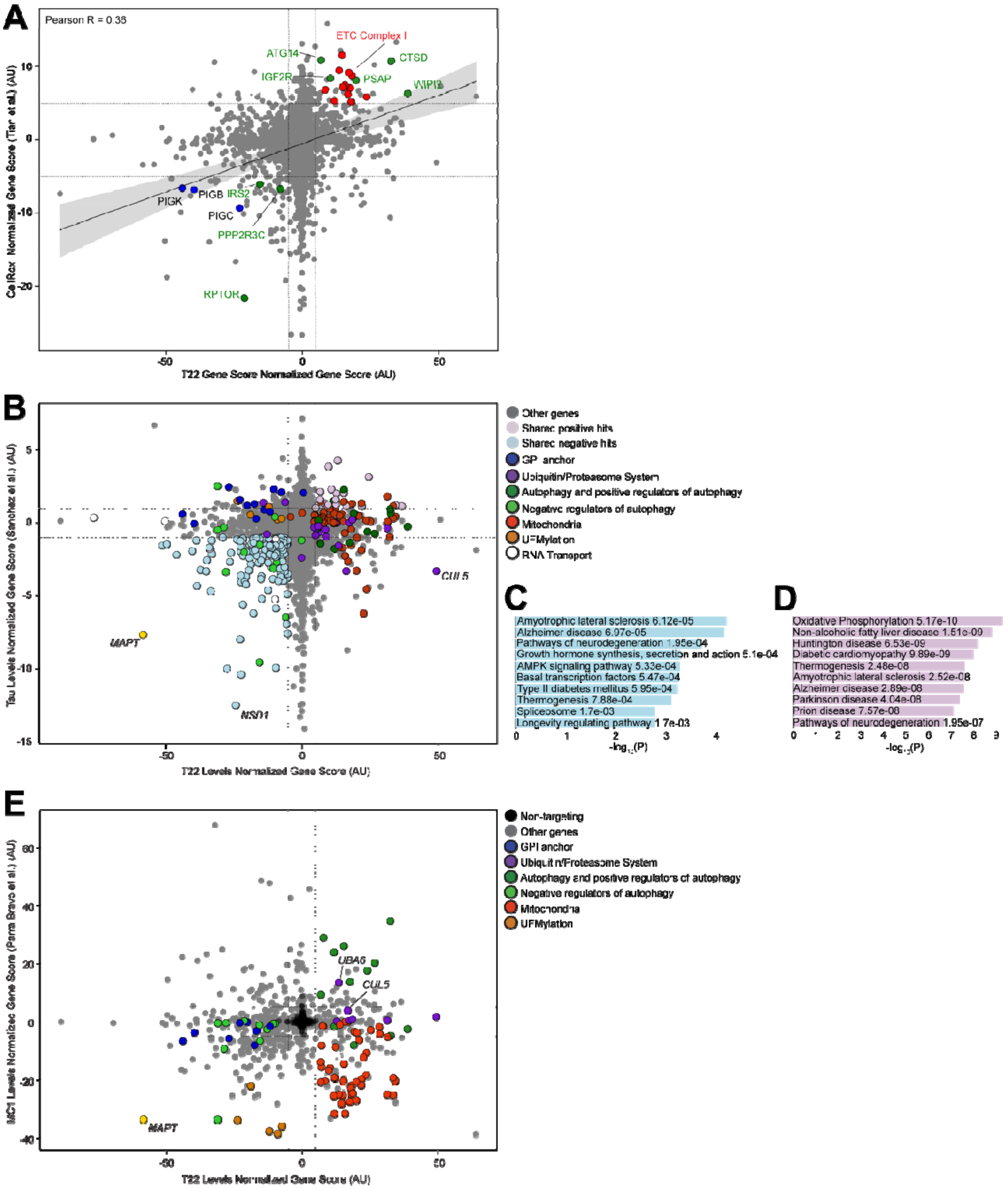

Figure S4: Comparison of screens in this work with others in the literature. (A) Shared positive and negative hits genes of Cell Rox (Tian et al.[59])and T22 screens (this work) were subjected to gene set enrichment analysis. Significant shared KEGG Pathways are labelled above. Red: ETC Complex I, Blue: GPI-anchor biosynthesis, Green: mTOR signaling and autophagy. Other genes involved in oxidative stress are labeled in black. **(B)** Comparison of primary screen with screen for tau levels (Sanchez et al[68]). **(C)** and **(D**) KEGG pathway analysis

bioRxiv preprint doi: https://doi.org/10.1101/2023.06.16.545386; this version posted October 26, 2024. The copyright holder for this preprint (which was not certified by peer review) is the author/funder, who has granted bioRxiv a license to display the preprint in perpetuity. It is made available under aCC-BY 4.0 International license.

of shared genes knockdown of which decrease (C) or increase (D) T22 levels (this work) or tau levels (Sanchez et al.). **(E)** Comparison of primary screen with screen for seeding induced tau aggregation (Parra Bravo et al.[70]).

bioRxiv preprint doi: https://doi.org/10.1101/2023.06.16.545386; this version posted October 26, 2024. The copyright holder for this preprint (which was not certified by peer review) is the author/funder, who has granted bioRxiv a license to display the preprint in perpetuity. It is made available under aCC-BY 4.0 International license.

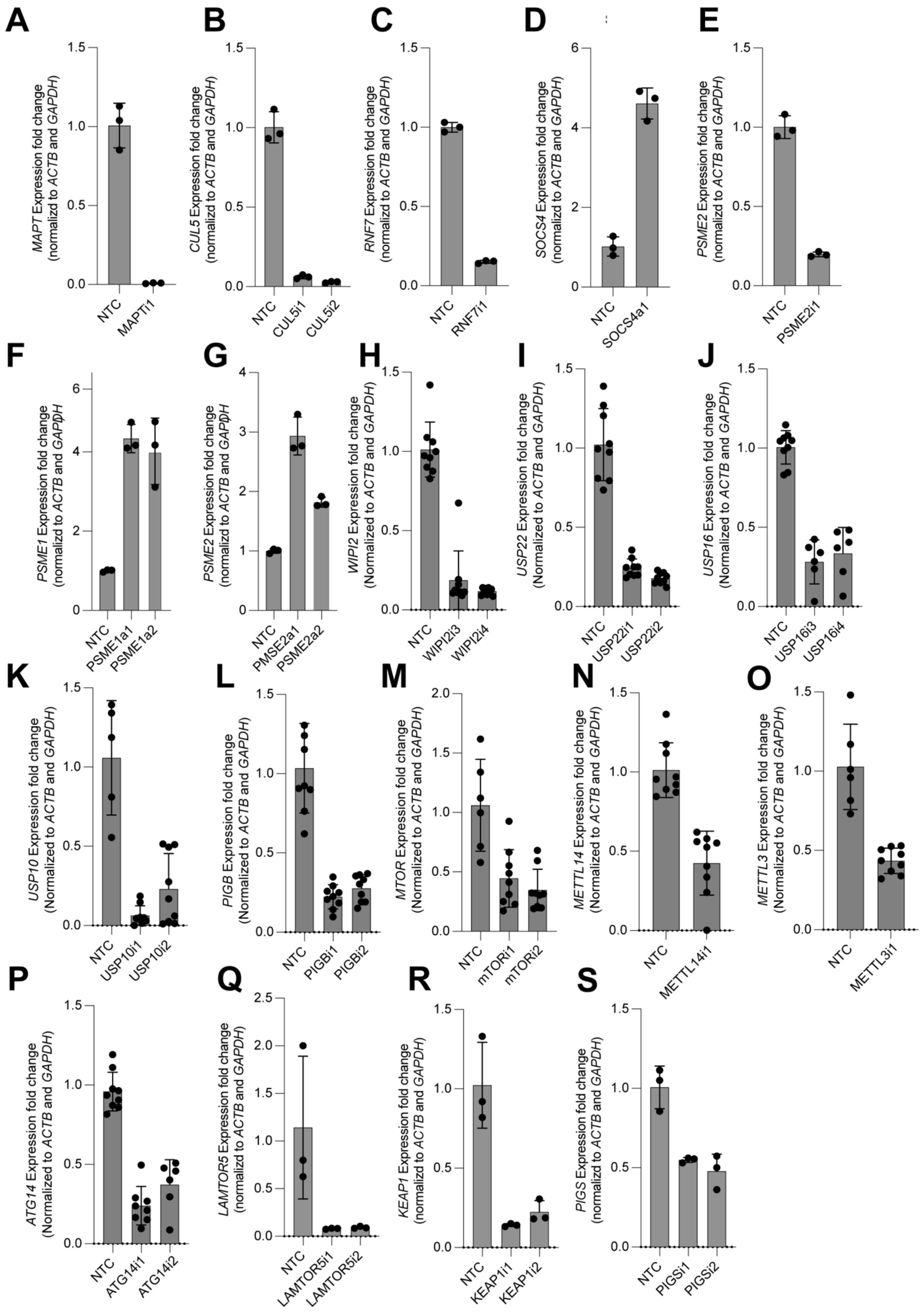

bioRxiv preprint doi: https://doi.org/10.1101/2023.06.16.545386; this version posted October 26, 2024. The copyright holder for this preprint (which was not certified by peer review) is the author/funder, who has granted bioRxiv a license to display the preprint in perpetuity. It is made available under aCC-BY 4.0 International license.

Figure S5: qPCR-based quantification effect of sgRNAs used in this study on expression of the targeted genes. (A) sgRNAs targeting *MAPT* in CRISPRi. **(B)** sgRNAs targeting *CUL5* in CRISPRi. **(C)** sgRNAs targeting *RNF7* in CRISPRi. **(D)** sgRNAs targeting *SOCS4* in CRISPRa. **(E)** sgRNAs targeting *PSME2* in CRISPRi. **(F)** sgRNAs targeting *PSME1* in CRISPRa. **(G)** sgRNAs targeting *PSME2* in CRISPRa. **(H)** sgRNAs targeting *WIPI2* in CRISPRi. **(I)** sgRNAs targeting *USP22* in CRISPRi. **(J)** sgRNAs targeting *USP16* in CRISPRi. **(K)** sgRNAs targeting *USP10* in CRISPRi. **(L)** sgRNAs targeting *PIGB* in CRISPRi. **(M)** sgRNAs targeting *MTOR* in CRISPRi. **(N)** sgRNAs targeting *METTL14* in CRISPRi. **(O)** sgRNAs targeting *METTL3* in CRISPRi. **(P)** sgRNAs targeting *ATG14* in CRISPRi. **(Q)** sgRNAs targeting *LAMTOR5* in CRISPRi. **(R)** sgRNAs targeting *KEAP1* in CRISPRi. **(S)** sgRNAs targeting *PIGS* in CRISPRi. (A)-(G), (Q)-(R) Average of three biological replicates, (H)-(P), Average of nine biological replicates. For (K), (M), (O), NTCs are average of six biological replicates. For all samples, error bars are ±standard deviation.

bioRxiv preprint doi: https://doi.org/10.1101/2023.06.16.545386; this version posted October 26, 2024. The copyright holder for this preprint (which was not certified by peer review) is the author/funder, who has granted bioRxiv a license to display the preprint in perpetuity. It is made available under aCC-BY 4.0 International license.

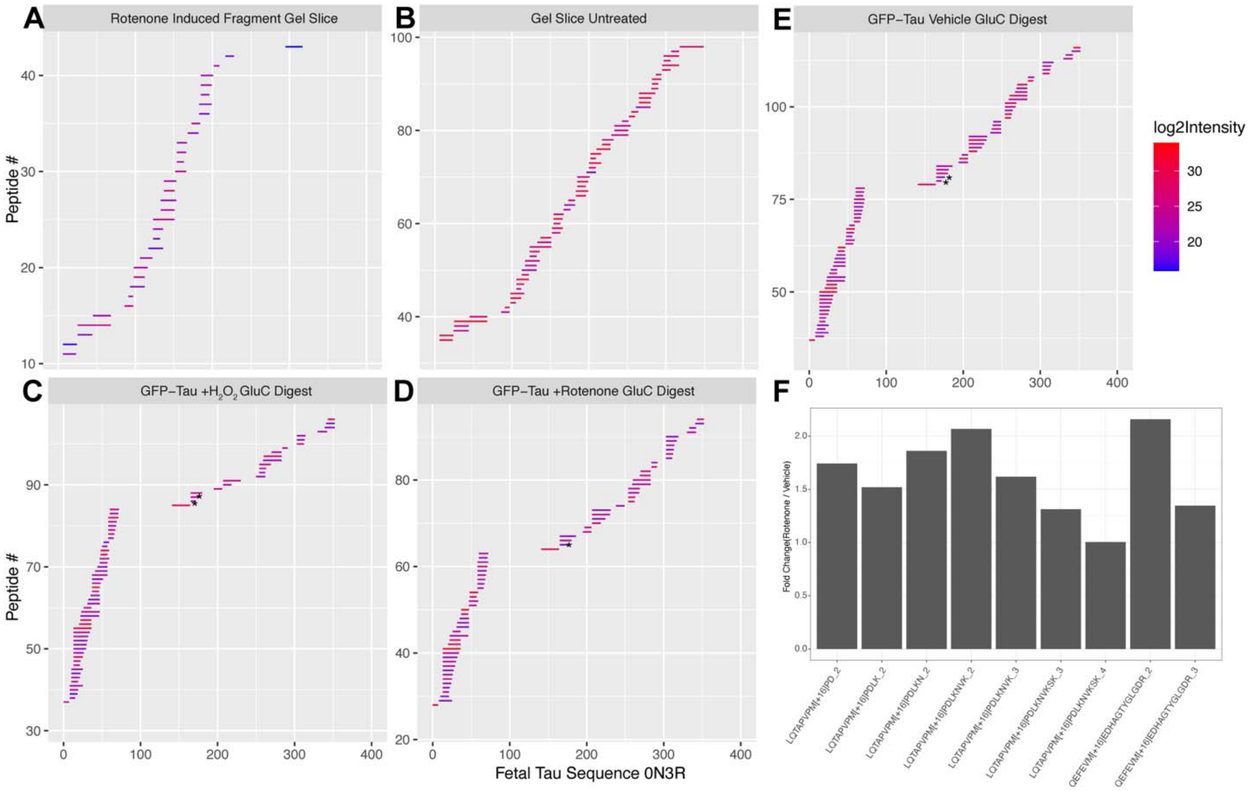

Figure S6– Plots of peptides and their intensities from mass spectrometry experiments. (A) Rotenone induced fragment excised from SDS/PAGE gel. **(B)** Full length tau excised from SDS/PAGE gel **(C)** Purified tau peptides from hydrogen peroxide treated neurons digested with GluC. **(D)** Purified tau peptides from 200nM rotenone treated neurons digested with GluC. **(E)** Purified tau peptides from vehicle treated neurons digested with GluC. Stars denote neo-tryptic termini. **(F)** Fold change intensities of oxidized methionine peptides measure by mass spectrometry. Average of four replicates for each peptide.

Supplemental Table 1 – qPCR primers and sgRNA protospacer sequences used in this study.
Supplemental Table 2 – Screen results for primary and secondary screens. Each screen is in a separate tab.
Supplemental Table 3 – All hit genes for primary and secondary screens. Each screen is in a separate tab.
Supplemental Table 4 – sgRNA protospacers sequences for secondary library
Supplemental Table 5 – All identified ubiquitination sites for *in vitro* ubiquitination. Each substrate is in a separate tab.
Supplemental Table 6 – All correlation scores calculated from the SEA-AD dataset.
