# Supplementary material for: CRISPR screens in iPSC-derived neurons reveal principles of tau proteostasis
Source: bioRxiv. 2023 Jun 26:2023.06.16.545386. Preprint. [Version 2] doi: 10.1101/2023.06.16.545386 (PMC10312804; doi:10.1101/2023.06.16.545386)
Supplement: Supplementary file 1 [file NIHPP2023.06.16.545386v2-supplement-5.pdf]

bioRxiv preprint doi: https://doi.org/10.1101/2023.06.16.545386; this version posted June 26, 2023. The copyright holder for this preprint (which was not certified by peer review) is the author/funder, who has granted bioRxiv a license to display the preprint in perpetuity. It is made available under aCC-BY 4.0 International license.

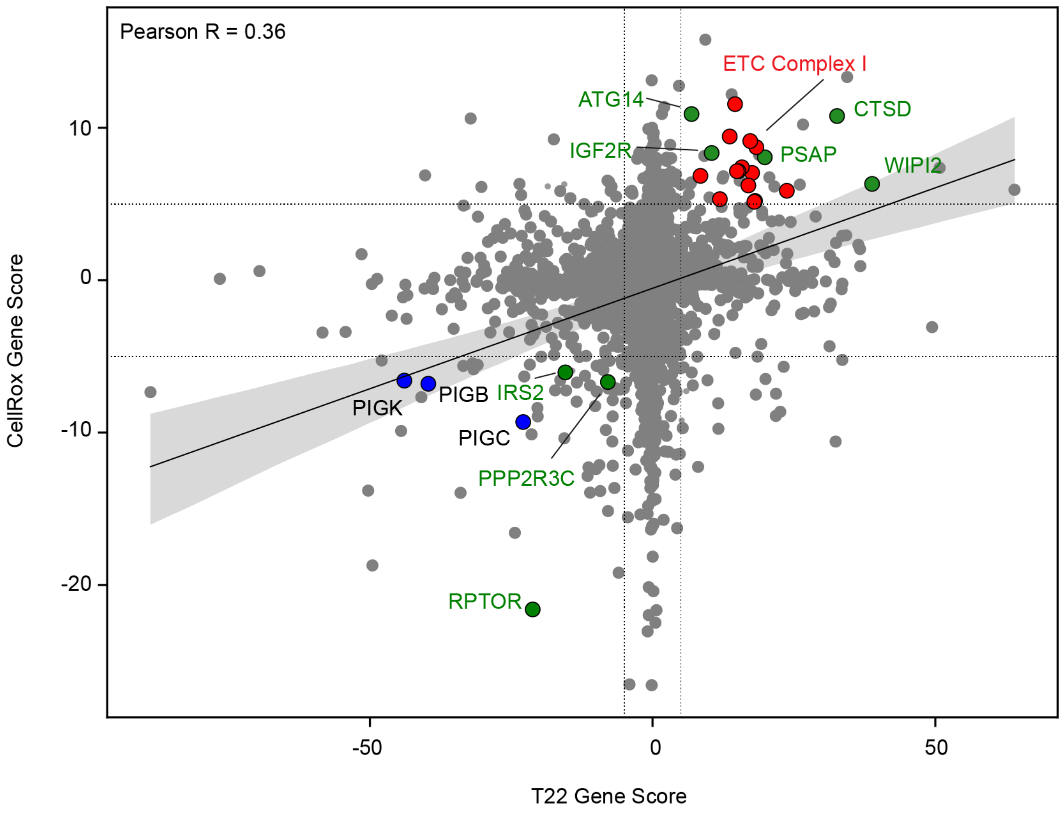

Figure S1 – Comparison between T22 and CellRox genome-wide CRISPRi screens. Gene set enrichment was performed for shared positive and negative hits using ENRICHR and genes in identified enriched pathways are labelled.

bioRxiv preprint doi: https://doi.org/10.1101/2023.06.16.545386; this version posted June 26, 2023. The copyright holder for this preprint (which was not certified by peer review) is the author/funder, who has granted bioRxiv a license to display the preprint in perpetuity. It is made available under aCC-BY 4.0 International license.

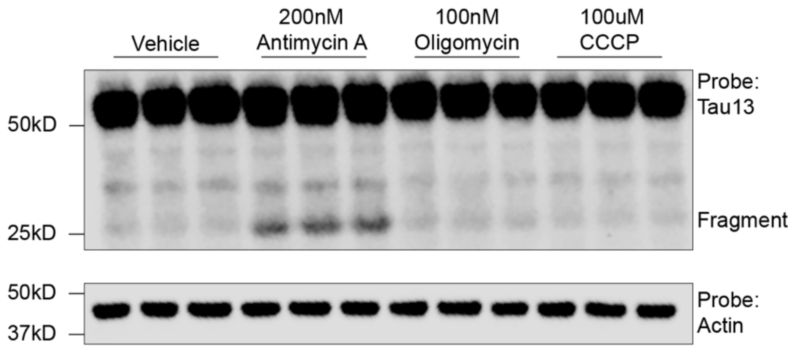

Figure S2 – Inhibition of ETC complex III, but not complex V or uncoupling of the mitochondrial protein gradient promotes fragment formation. Western blot of vehicle treated, antimycin A treated (complex III inhibitor), oligomycin treated (complex V inhibitor) or CCCP treated (protein gradient uncoupler). Data are three biological replicates.

bioRxiv preprint doi: https://doi.org/10.1101/2023.06.16.545386; this version posted June 26, 2023. The copyright holder for this preprint (which was not certified by peer review) is the author/funder, who has granted bioRxiv a license to display the preprint in perpetuity. It is made available under aCC-BY 4.0 International license.

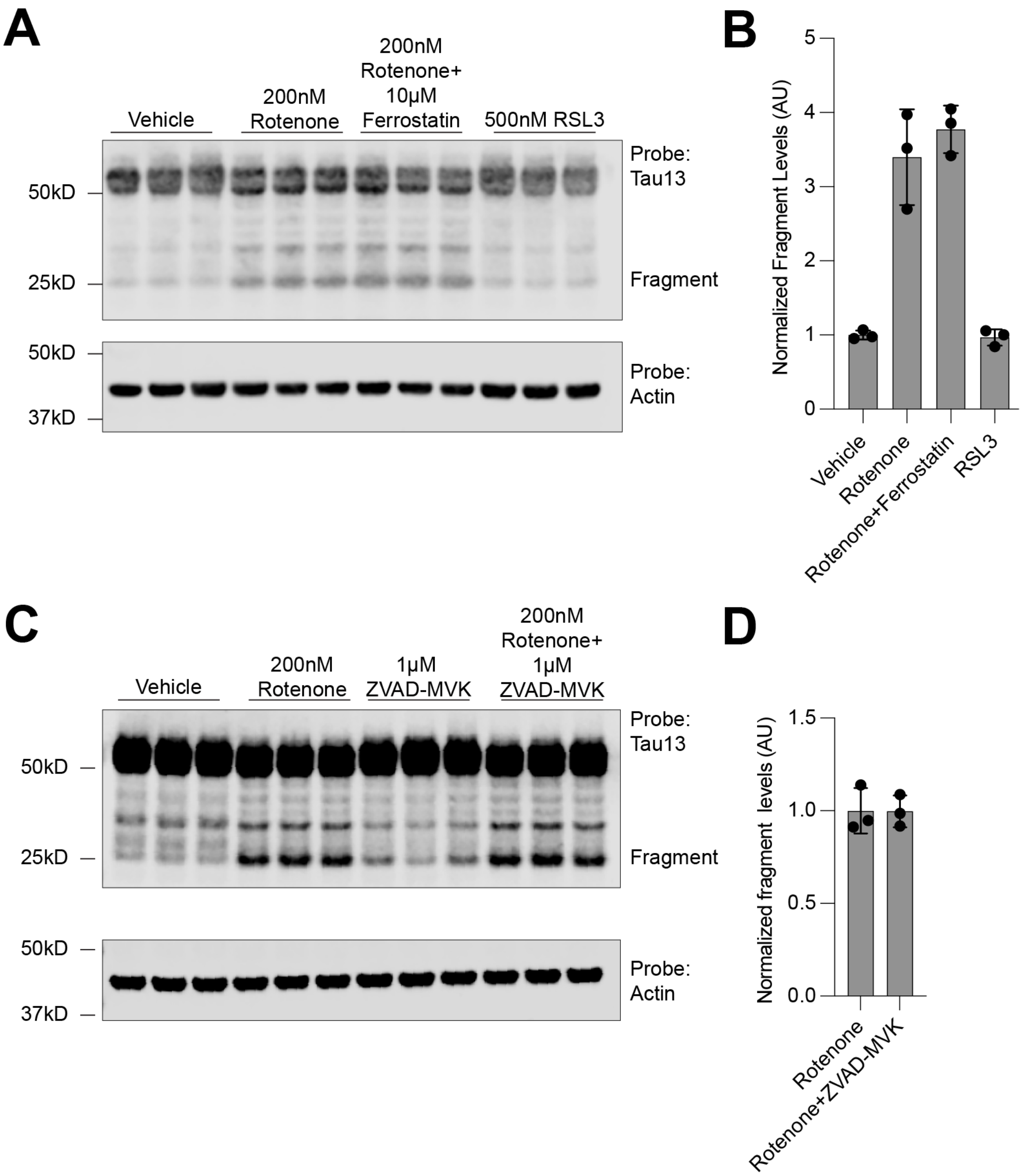

Figure S3 – Apoptosis and ferroptosis do not control fragment formation. (A) Western blot of rotenone-treated neurons with the ferroptosis inhibitor ferrostatin, or ferroptosis promoting molecule RSL3 reveals no changes to tau cleavage fragments. **(C)** Quantitation of data in (A). Western blot of rotenone-treated neurons with the pan-caspase inhibitor ZVAD-MVAK reveals no changes to tau cleavage fragments. **(D)** Quantitation of data in (C). Data are average of three biological replicates.

bioRxiv preprint doi: https://doi.org/10.1101/2023.06.16.545386; this version posted June 26, 2023. The copyright holder for this preprint (which was not certified by peer review) is the author/funder, who has granted bioRxiv a license to display the preprint in perpetuity. It is made available under aCC-BY 4.0 International license.

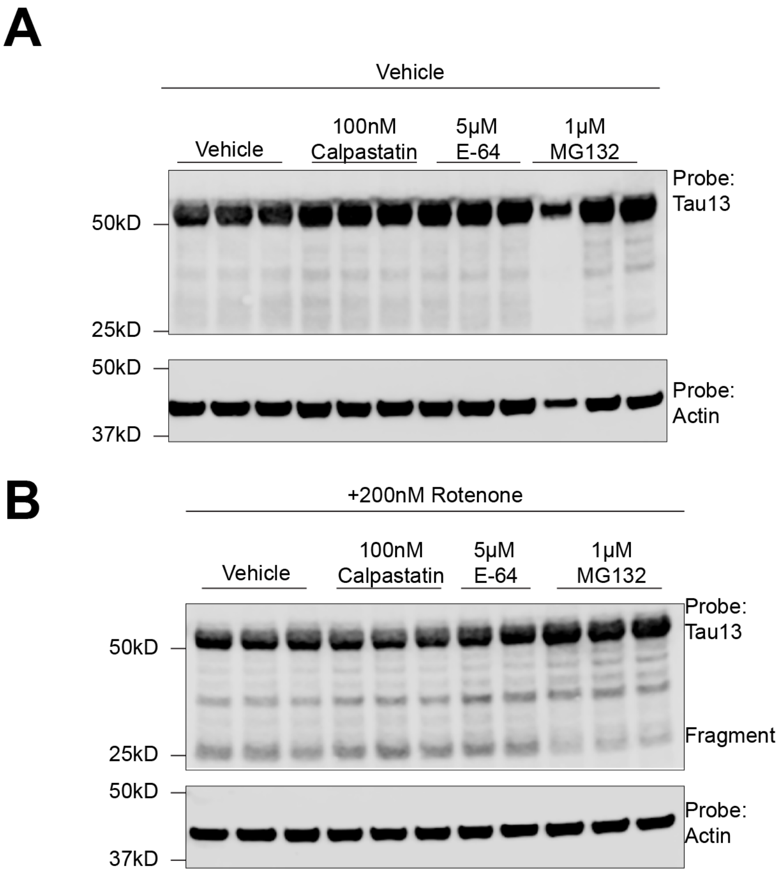

Figure S4 – Proteasome inhibition decreases fragment formation. (A) Western blot of neurons with the cathepsin inhibitor E64, calpain inhibitor calpastatin, and proteasome inhibitor MG132. **(B)** Western blot of rotenone treated neurons with the cathepsin inhibitor E64, calpain inhibitor calpastatin, and proteasome inhibitor MG132.

bioRxiv preprint doi: https://doi.org/10.1101/2023.06.16.545386; this version posted June 26, 2023. The copyright holder for this preprint (which was not certified by peer review) is the author/funder, who has granted bioRxiv a license to display the preprint in perpetuity. It is made available under aCC-BY 4.0 International license.

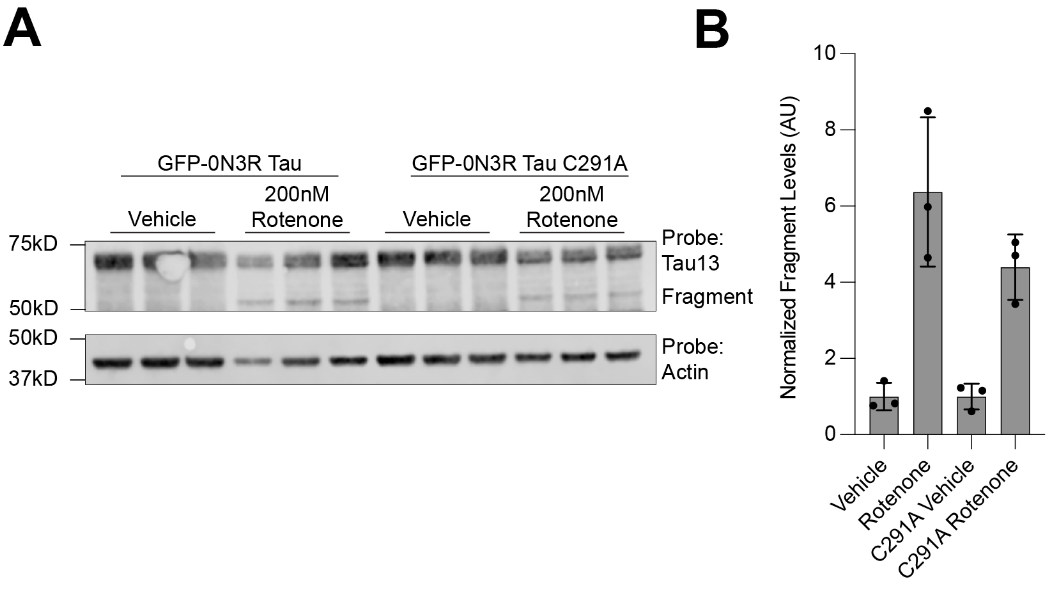

Figure S5 – Mutation of Tau C291 does not affect fragment formation. (A) Western blot of neurons transduced with GFP-tau or GFP-tau C291A treated with rotenone or vehicle. **(B).** Quantitation of data in (A). All samples are the average of three biological replicates, error bars are ±standard deviation.

bioRxiv preprint doi: https://doi.org/10.1101/2023.06.16.545386; this version posted June 26, 2023. The copyright holder for this preprint (which was not certified by peer review) is the author/funder, who has granted bioRxiv a license to display the preprint in perpetuity. It is made available under aCC-BY 4.0 International license.

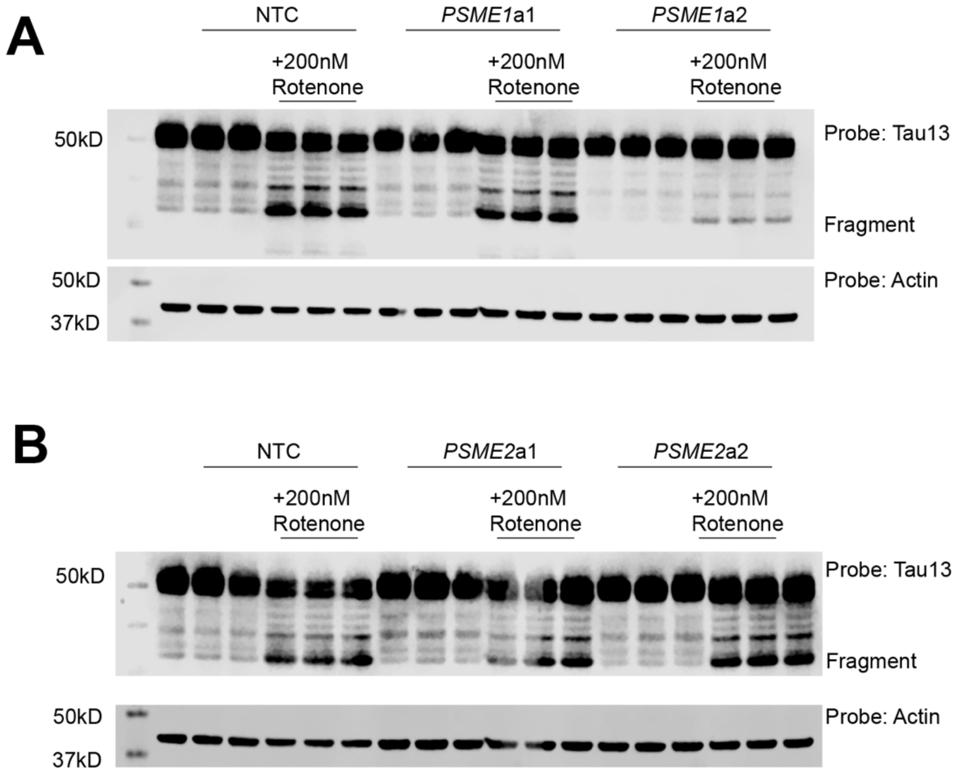

Figure S6 – Gels quantified in Figure 6 (A) CRISPRa of PSME1.**(B)** CRISPRa of PSME2.

bioRxiv preprint doi: https://doi.org/10.1101/2023.06.16.545386; this version posted June 26, 2023. The copyright holder for this preprint (which was not certified by peer review) is the author/funder, who has granted bioRxiv a license to display the preprint in perpetuity. It is made available under aCC-BY 4.0 International license.

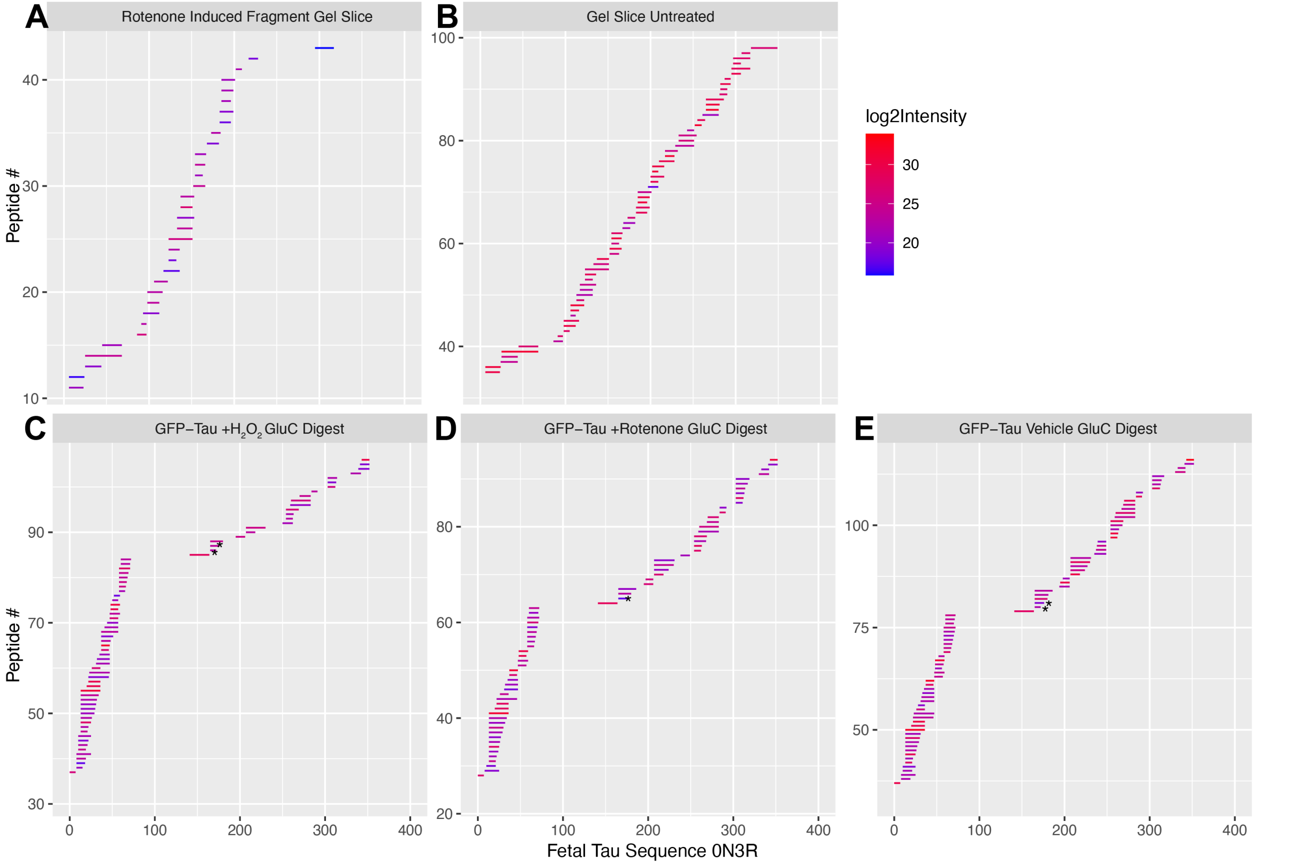

Figure S7 – Plots of peptides and their intensities from mass spectrometry experiments. (A) Rotenone induced fragment excised from SDS/PAGE gel. **(B)** Full length tau excised from SDS/PAGE gel **(C)** Purified tau peptides from hydrogen peroxide treated neurons digested with GluC. **(D)** Purified tau peptides from 200nM rotenone treated neurons digested with GluC. **(E)** Purified tau peptides from vehicle treated neurons digested with GluC. Stars denote neo-tryptic termini.

bioRxiv preprint doi: https://doi.org/10.1101/2023.06.16.545386; this version posted June 26, 2023. The copyright holder for this preprint (which was not certified by peer review) is the author/funder, who has granted bioRxiv a license to display the preprint in perpetuity. It is made available under aCC-BY 4.0 International license.

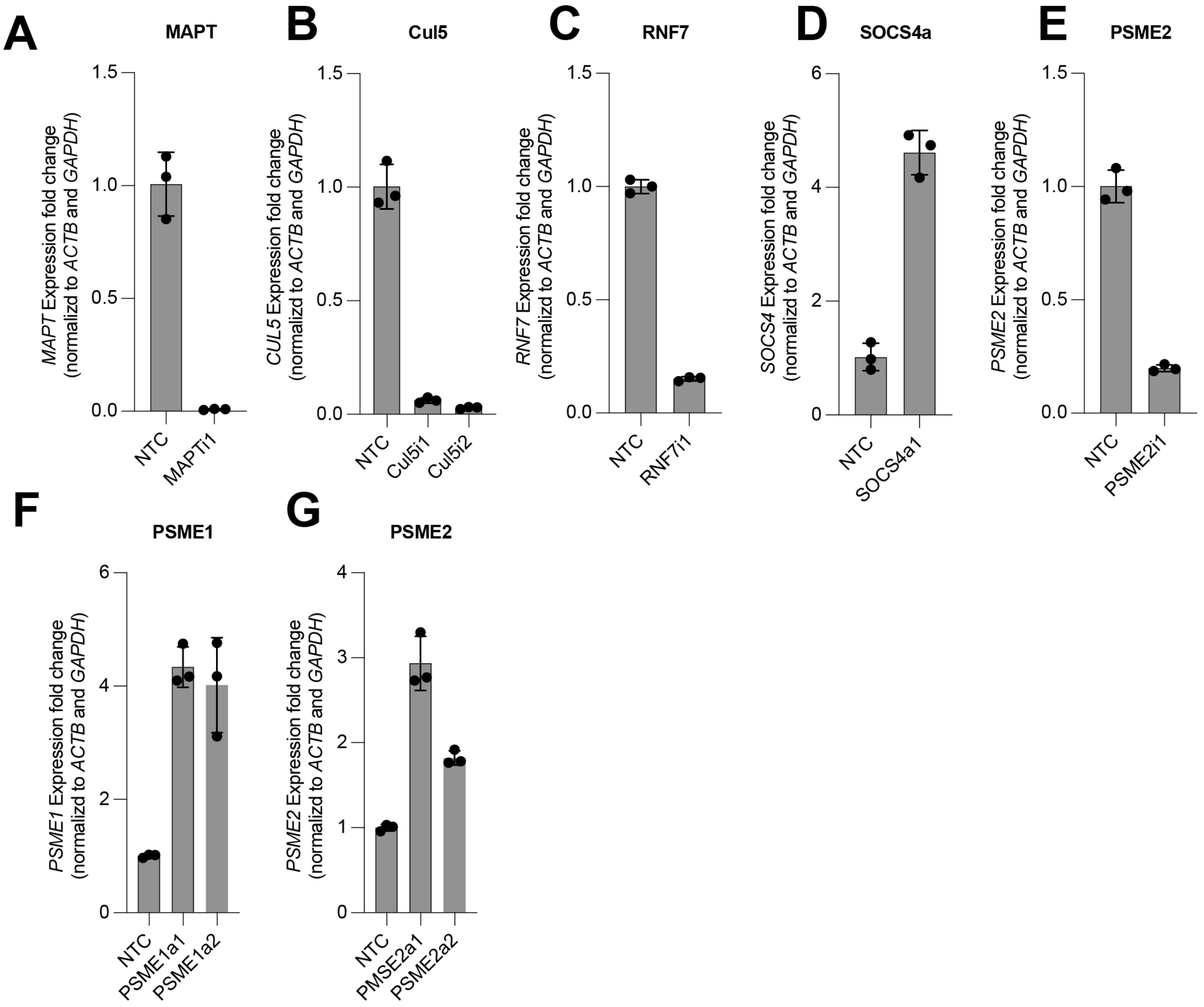

Figure S8 – qPCR-based quantification effect of sgRNAs used in this study on expression of the targeted genes. (A) sgRNAs targeting *MAPT* in CRISPRi. **(B)** sgRNAs targeting *CUL5* in CRISPRi. **(C)** sgRNAs targeting *RNF7* in CRISPRi. **(D)** sgRNAs targeting *SOCS4* in CRISPRa. **(E)** sgRNAs targeting *PSME2* in CRISPRi. **(F)** sgRNAs targeting *PSME1* in CRISPRa. **(G)** sgRNAs targeting *PSME2* in CRISPRa. All samples are the average of three biological replicates, error bars are ±standard deviation.

bioRxiv preprint doi: https://doi.org/10.1101/2023.06.16.545386; this version posted June 26, 2023. The copyright holder for this preprint (which was not certified by peer review) is the author/funder, who has granted bioRxiv a license to display the preprint in perpetuity. It is made available under aCC-BY 4.0 International license.

Supplemental Table Legends

Table S1 – qPCR primers and sgRNA protospacer sequences used in this study.

Table S2 – Screen results for primary and secondary screens. Each screen is in a separate tab. First column is gene. Second column is phenotype (log2 fold change of high bin over low bin). Third column is p-value. Fourth column is gene score (phenotype times -log10(p-value)). See methods for determination of phenotype and p-value.

Table S3 – All hit genes for primary and secondary screens. Each screen is in a separate tab. First column is gene. Second column is phenotype (log2 fold change of high bin over low bin). Third column is p-value. Fourth column is gene score (phenotype times -log10(p-value)). See methods for determination of phenotype and p-value.

Table S4 – sgRNA protospacers sequences for secondary screen library
